# Competencies and pedagogical foundations for faculty development in interprofessional health education: a scoping review*

**DOI:** 10.1590/1980-220X-REEUSP-2024-0421en

**Published:** 2025-11-03

**Authors:** Daniella Rosaly Leite, Patrícia Silva Carneiro, Vivian Aline Mininel, Rosana Aparecida Salvador Rossit, Valéria Marli Leonello, Geisa Colebrusco de Souza Gonçalves, Jaqueline Alcântara Marcelino da Silva

**Affiliations:** 1 Universidade Federal de São Carlos, São Carlos, SP, Brazil.; 2 Universidade Federal de Goiás, Goiânia, GO, Brazil.; 3 Universidade Federal de São Paulo, São Paulo SP, Brazil.; 4 Universidade de São Paulo, São Paulo SP, Brazil.

**Keywords:** Professional Competence, Competency-Based Education, Teacher Training, Faculty, Interprofessional Education

## Abstract

**Objective:**

To map the competencies for faculty development in interprofessional health education.

**Method:**

This is a scoping review based on the recommendations of the Joanna Briggs Institute with the guiding question: “What competencies are used for faculty development in interprofessional education?” Searches were conducted in the MEDLINE/OVID, ERIC, CINAHL, Scopus, Web of Science, Google Scholar, EBSCO Open Dissertations, BDTD, CAPES Theses and Dissertations, OpenGrey, and MedNar databases, with no time restrictions and in Portuguese, Spanish, and English.

**Results:**

Twenty-six publications were included, grouped into two categories: the first refers to interprofessional collaborative and facilitation competencies for interprofessional education, with an emphasis on the domains of communication and teamwork. The second category concerns the pedagogical foundations of IPE, theories, approaches, teaching-learning and assessment strategies in faculty development actions for interprofessional education.

**Conclusion:**

The evidence mapped contributes to strengthening faculty development initiatives and advancing the theoretical and methodological foundations of interprofessional health education.

## INTRODUCTION

Healthcare organizations have been dealing with challenges caused by the fragmentation of healthcare systems and care practices that compromise the availability, access, and quality of services, as well as the difficulties inherent in the training of healthcare professionals. There is an urgent need for innovative strategies, with an emphasis on the development of policies and programs that consolidate training and work in the health field^(1,2)^, since health services require professionals prepared with interprofessional collaborative competencies to deal with changes and challenges in health care^(2)^.

Interprofessional health education (IPE) seeks to contribute to the reformulation of undergraduate and graduate course curricula, with the aim of preparing professionals to work efficiently in teams, focusing on interaction, integration, and collaboration^(3)^, with care centered on the person, group, or population^(4)^. IPE is a global movement, and in the Brazilian context, it finds possibilities for implementation in the services of the Unified Health System (SUS), in different training spaces: undergraduate, residency, and continuing education in health. In these educational contexts, IPE contributes to mobilizing reflections on the work process and co-production aimed at strengthening interprofessional collaborative skills and practices committed to the health needs of users and the population, as protagonists of care^(5)^.

With the expansion of IPE, it is crucial to prepare teachers to deal with the challenges of this innovative educational approach^(2,6)^. Teachers are required to lead, facilitate, and develop IPE curricula, but they find it difficult to take on these duties and responsibilities^(7)^ because they have not been exposed to interprofessional learning activities and practices in their professional training or teaching practice^(8)^, with few faculty development initiatives, especially from an IPE perspective.

Although the term faculty is widely used for university professors, they are not the only agents involved in health education. There is a growing understanding that professionals working in the healthcare network should also participate training to develop IPE activities, with recognition of their contribution in professional education^(9)^. Thus, it is recognized that IPE initiatives involve facilitators who are health professionals, doctors, masters, training specialists, preceptors, tutors, and supervisors, who act as agents jointly responsible for health training and need training opportunities to work in health education.

A systematic review study indicated the need to adopt initiatives for faculty development in IPE, since among the main aspects for successful interprofessional intervention are the ability of faculty to facilitate, understanding of the educational approach, teaching-learning strategies, evaluation processes, and competencies in IPE^(10)^. Similarly, a study pointed out that IPE practice emphasized interprofessional collaborative competencies for students, but teaching facilitation competencies were neglected^(11)^.

Thus, the success of an educational intervention requires learning objectives, expected outcomes, content, teaching-learning and assessment strategies, as well as the skills to be developed in a coherent manner^(12)^. In order for curriculum planning, implementation, and assessment to be anchored in IPE, teachers need opportunities to develop new skills^(7,10,13)^.

Given the above and in the absence of previous studies, we identified the need to map the competencies for faculty development in IPE, with the intention of minimizing the gaps found in the literature, guiding policies and programs in the local (institutional) context and in the macro context of health education (system), to support future initiatives on the topic. The objective of this scoping review was to map the competencies for faculty development in IPE.

## THEORETICAL FRAMEWORK

In the literature, there are two key frameworks describing interprofessional collaborative competencies, one Canadian^(14)^ and one American^(15)^, although they do not address faculty development, they indicate competencies for professionals working in health systems. The framework of the Canadian Interprofessional Health Collaborative (CIHC)^(14)^ encompasses four competencies: clarity of roles; team functioning; interprofessional conflict resolution; and collaborative leadership, dependent on two main domains of competencies: interprofessional communication and user, family, and community-centered care.

The Canadian framework aims to identify the knowledge, skills, attitudes, behaviors, and judgments necessary to achieve excellence and apply them in situations of interprofessional collaboration. Thus, these competencies can contribute to the planning of IPE actions, developed by teachers and other facilitators^1^, aiming at successful educational actions^(14)^.

In 2024, the CIHC framework was updated, with changes in terminology aimed at improving interprofessional collaboration. Among the changes, the inclusion of healthcare and health service partners, as well as all people involved in care relationships, stands out. The purpose of the update was to improve the health care provided through collaborative and relationship-centered partnerships and to facilitate joint decision-making about social and health needs. The framework is based on factors that influence the application of competencies, such as inclusion, access, and equity, related to the complexity and context of health care and services^(4)^.

The US framework, Interprofessional Education Collaborative (IPEC)^(15)^, presents essential competencies for collaborative practice. Published in 2011, with four thematic areas related to IPE: values and ethics; roles and responsibilities; interprofessional communication; team and teamwork^(15)^, it was updated in 2016, highlighting the change of presenting the four thematic areas mentioned in a single domain: interprofessional collaborative competence, with an emphasis on the centrality of care for users, families, and communities^(16)^.

The latest version of the IPEC framework published in 2023^(17)^ expresses collaborative interprofessional practice (CIP) as essential for achieving better health outcomes from the perspective of safe, quality, accessible, equitable, and person-centered care. The single domain of interprofessional collaboration competence and the four areas (values and ethics; roles and responsibilities; communication; teams and teamwork) were maintained, with an indication of the commitment to lifelong health training, aiming at engaging newly graduated and experienced health professionals in the construction of these competencies.

## METHOD

### Type of Study

This is a scoping review based on the recommendations of the Joanna Briggs Institute (JBI) and the *Preferred Reporting Items for Systematic Reviews and Meta-Analysis Extension for Scoping Reviews* (PRISMA) - *Extension for Scoping Reviews* (PRISMA-ScR): *Checklist and Explanation* ^(18)^. The scoping review aims to map the main concepts that support a given area of knowledge, examine the extent and scope of the research object, substantiate and disseminate the data, and identify existing gaps^(19)^. The protocol was developed and reviewed by the authors and registered prospectively on the Open Science Framework (OSF) plataform: https://osf.io/6gazx/. The study was conducted in sequential stages: identification of the research question, extraction, analysis, and presentation of results^(19)^. During the process, the authors held discussions to achieve alignment and consensus. 

### Research Question

The Population, Concept, and Context (PCC) mnemonic strategy was used to construct the research question. This review had as its population (P) faculty preceptors, tutors, mentors, professors, or facilitators of higher education in health sciences courses; the concept (C) was competencies related to faculty development for IPE; and the context (C) was universities and/or health training practice settings. Thus, the following guiding question was adopted: “What competencies are used for faculty development in IPE?”

### Eligibility Criteria

Theoretical studies (theoretical essays and literature reviews), primary and secondary empirical studies, quantitative, mixed qualitative, and quasi-experimental studies published in Portuguese, Spanish, and English, without time restrictions, with a population composed of higher education teachers in the health sciences and field preceptors who addressed the competencies necessary for faculty development in the context of IPE. Therefore, in this review, we considered, in addition to faculty, all health professionals involved in student training (doctors, masters, specialists, bachelors), preceptors, supervisors, and facilitators who participate in health training. Gray literature, such as dissertations, theses, books, and/or book chapters and manuals, was included when it answered the research question. The exclusion criteria included reviews, editorials, conference proceedings, and events.

### Data Sources And Search Strategy

The search strategy was developed by the study authors with the support of an experienced librarian, based on a combination of Health Sciences Descriptors (DeCS) and Medical Subject Headings (MeSH), and Boolean operators AND and OR, presented in Chart 1. The searches were conducted in December 2023 in the following databases: Medical Literature Analysis and Retrieval System (MEDLINE/OVID), Education Resources Information Center (ERIC), Cumulative Index to Nursing and Allied Health Literature (CINAHL) via EBSCO, Web of Science Institute for Scientific Information, Scopus SciVerse, Google Scholar, EBSCO *Open Dissertations*, Thesis and Dissertation Catalog (CTD) of the Coordination for the Improvement of Higher Education Personnel (CAPES), Brazilian Digital Library of Theses and Dissertations (BDTD), *Deep Web Search Engine* (MedNar), and *System for Information on Grey Literature in Europe* (OpenGrey). To identify other eligible studies, manual searches were conducted in the references of the selected studies.

**Chart 1:** Database search strategy – São Carlos, SP, Brazil, 2024.

Database	Expression
MEDLINE/OVID	**S1** (Faculty* OR Mentor* OR Professor* OR Tutor* OR Teacher* OR Preceptor* OR Instructor* OR Facilitat* OR Development).mp. [mp = title, abstract, original title, name of substance word, subject heading word, floating sub-heading word, keyword heading word, organism supplementary concept word, protocol supplementary concept word, rare disease supplementary concept word, unique identifier, synonyms] **S2** (Competency Based Education OR Competency Based Education OR Education, Competency-Based OR Competency-Based Educations OR Education, Competency Based OR Educations, Competency-Based OR Competency).mp. [mp = title, abstract, original title, name of substance word, subject heading word, floating sub-heading word, keyword heading word, organism supplementary concept word, protocol supplementary concept word, rare disease supplementary concept word, unique identifier, synonyms] **S3** (Interprofessional education OR interprofessional education OR IPE OR multi-professional education OR multi-professional education OR multi-professional education OR multidisciplinary education OR multi-disciplinary education).mp. [mp = title, abstract, original title, name of substance word, subject heading word, floating sub-heading word, keyword heading word, organism supplementary concept word, protocol supplementary concept word, rare disease supplementary concept word, unique identifier, synonyms] **S4** (1 and 2 and 3) ((Faculty* OR Mentor* OR Professor* OR Tutor* OR Teacher* OR Preceptor* OR Instructor* OR Facilitat* OR Development) AND (Competency Based Education OR Competency Based Education OR Education, Competency-Based OR Competency-Based Educations OR Education, Competency Based or Educations, Competency-Based OR Competency) AND (Interprofessional education OR interprofessional education OR IPE OR multi-professional education OR multi-professional education OR multi-professional education or multidisciplinary education OR multi-disciplinary education)).mp. [mp = title, book title, abstract, original title, name of substance word, subject heading word, floating sub-heading word, keyword heading word, organism supplementary concept word, protocol supplementary concept word, rare disease supplementary concept word, unique identifier, synonyms, population supplementary concept word, anatomy supplementary concept word]
ERIC and CINAHL	**S1** (Faculty* OR Mentor* OR Professor* OR Tutor* OR Teacher* OR Preceptor* OR Instructor* OR Facilitat* OR Development) AND (Competency Based Education OR Competency Based Education OR Education, Competency-Based OR Competency-Based Educations OR Education, Competency Based OR Educations, Competency-Based OR Competency) AND (Interprofessional education OR interprofessional education OR IPE OR multi-professional education OR multi-professional education OR multi-professional education OR multidisciplinary education OR multi-disciplinary education)
Web of Science	**S1** (Faculty* OR Mentor* OR Professor* OR Tutor* OR Teacher* OR Preceptor* OR Instructor* OR Facilitat* OR Development) AND (“Competency-Based Education” OR “Competency Based Education” OR “Education, Competency-Based” OR “Competency-Based Educations” OR “Education, Competency Based” OR “Educations, Competency-Based” OR Competency) AND (“Interprofessional education” OR “interprofessional education” OR IPE OR “multiprofessional education” OR “multi-professional education” OR “multiprofessional education” OR “multidisciplinary education” OR “multi-disciplinary education”)
Scopus	**S1** TITLE-ABS-KEY Faculty* OR Mentor* OR Professor* OR Tutor* OR Teacher* OR Preceptor* OR Instructor* OR Facilitat* OR Development) AND (Competency Based Education OR Competency Based Education OR Education, Competency-Based OR Competency-Based Educations OR Education, Competency Based OR Educations, Competency-Based OR Competency) AND (Interprofessional education OR interprofessional education OR IPE OR multi-professional education OR multi-professional education OR multi-professional education OR multidisciplinary education OR multi-disciplinary education)
Google Scholar	**S1** (Faculty* OR Mentor* OR Professor* OR Tutor* OR Teacher* OR Preceptor* OR Instructor* OR Facilitat* OR Development) AND (Competency Based Education OR Competency Based Education OR Education, Competency-Based OR Competency-Based Educations OR Education, Competency Based OR Educations, Competency-Based OR Competency) AND (Interprofessional education OR interprofessional education OR IPE OR multi-professional education OR multi-professional education OR multi-professional education OR multidisciplinary education OR multi-disciplinary education)
EBSCO *Open Dissertations*	**S1** (Faculty* OR Mentor* OR Professor* OR Tutor* OR Teacher* OR Preceptor* OR Instructor* OR Facilitat* OR Development) AND (Competency Based-Education OR Competency Based Education OR Education, Competency-Based OR Competency-Based Educations OR Education, Competency Based OR Educations, Competency-Based OR Competency) AND (Interprofessional education OR interprofessional education OR IPE OR multi-professional education OR multi-professional education OR multi-professional education OR multidisciplinary education OR multi-disciplinary education)
BDTD/*Teses e dissertações da CAPES* (CAPES theses and dissertations)	**S1** “Professor AND Competências AND Educação Interprofissional”
MedNar	**S1** “Faculty AND Competency AND Interprofessional Education Development”
OpenGrey	**S1** “Faculty AND Interprofessional”

Source: Authors.

### Study Selection

The results obtained were exported to the *Rayyan®* ^(20)^ software to automatically remove duplicate studies. The screening and selection of studies were performed using a double-blind procedure by two independent reviewers in two stages, based on the selection of titles and abstracts in the first stage and the reading of the full texts in the second stage. Disagreements in the selection were resolved by a third reviewer. The reference lists of the studies selected in the second stage were also reviewed by the third reviewer to confirm the relevance of including the studies on the topic. The search results and selected studies are presented in the flowchart *Preferred Reporting Items for Systematic Reviews and Meta-Analyses Extension for Scoping Reviews* (PRISMA-ScR)^(18)^, shown below:

### Data Analysis And Summary Of Results

To extract data from the selected studies, a spreadsheet was created using Microsoft Excel® software. The following relevant information was selected from the studies: 1) Characterization: authorship, country, year, publication approach, population, and context; 2) Study identification, interprofessional collaborative competencies, teaching-learning theories or approaches, and evaluation process.

The data were analyzed using descriptive and deductive thematic content analysis^(19)^, which consisted of organizing the information in three stages: pre-analysis; exploration of the material; and interpretation of the results^(21)^, corresponding to the three stages of content analysis in scoping reviews: preparation, organization, and reporting^(19)^. Thus, the selected material was organized and coded into recording units (themes) and non- frequency indicators, followed by categorization and classification of themes by differentiation and regrouping^(21)^. The deductive analysis was based on the theoretical constructs of IPE and the competency frameworks of CIHC^(14)^ and IPEC^(15-17)^.

## RESULTS

**Figure 1:**
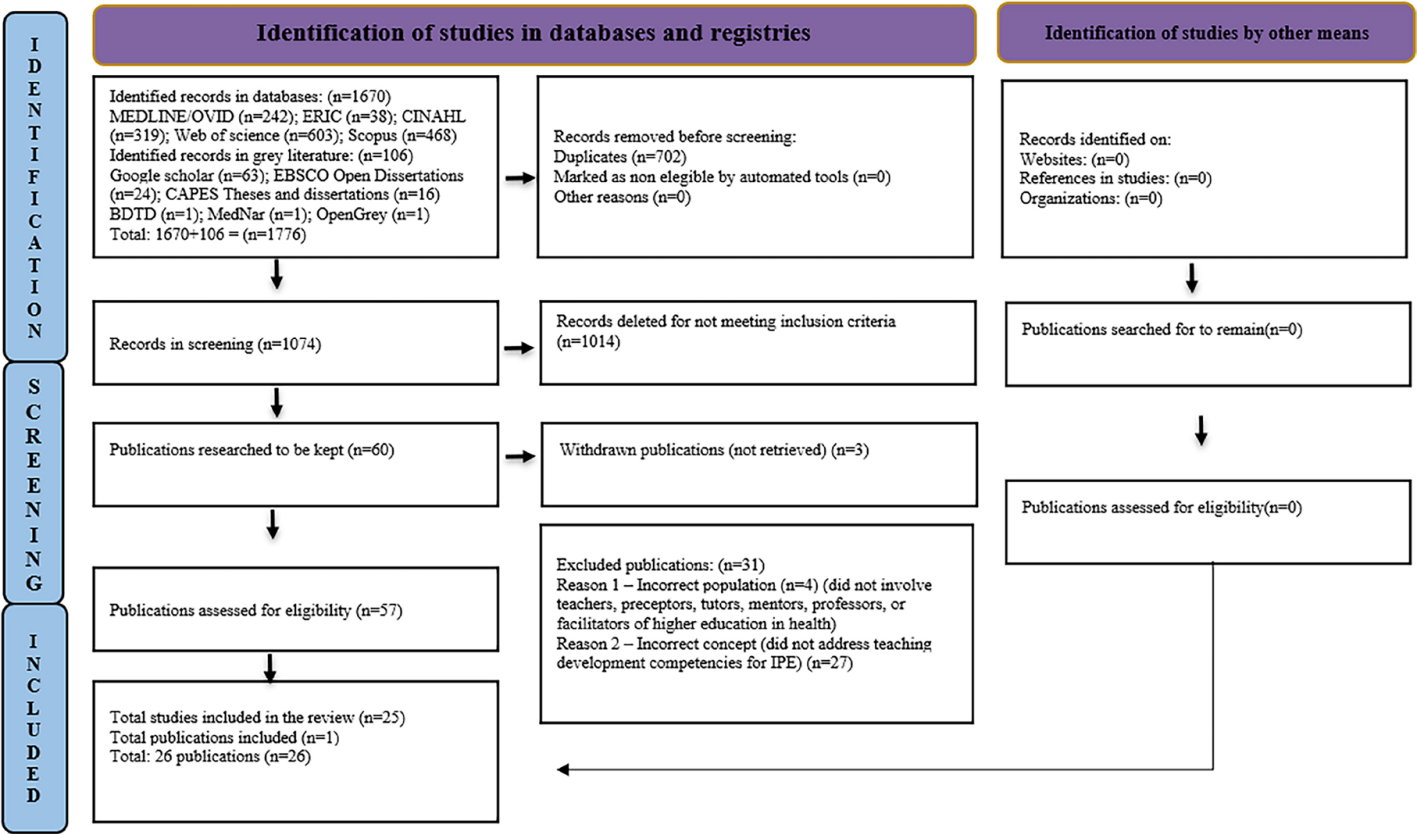
PRISMA-ScR flowchart, identification, screening, and selection of studies included in the scoping review – São Carlos, SP, Brazil, 2024.

**Source**: Author.

The searches totaled 1,776 records, 26 studies met the eligibility criteria and were included in this review as shown in Figure 1. They were published between 2009 and 2023, from the following countries: United States (n = 15; 57%), Canada (n = 3; 11%), South Africa (n = 2; 8%), Australia (n = 2; 8%), Germany (n = 1; 4%), Saudi Arabia (n = 1; 4%), Asia (n = 1; 4%), and India (n = 1; 4%). Most were mixed-method studies (n = 8; 31%), qualitative (n = 5; 19%), quantitative (n = 4; 15%), quasi-experimental (n = 4; 15%), narrative literature reviews (n = 3; 12%), and theoretical essays (n = 2; 8%).

Regarding the context of the studies, 11 (42%) were conducted in higher education institutions (HEIs), 10 (39%) in health institutions and HEIs, one (4%) in the South African Interprofessional Education Network, and four (15%) had no defined context, as they were theoretical essays or narrative reviews.

The participants in the studies included faculty, teachers, clinical supervisors, and preceptors from practice settings, health professionals involved in training, and interprofessionality specialists working in the health sciences. The data extracted from the publications are presented in Chart 2.

**Chart 2:** Characterization of studies according to author, country, year of publication, publication approach, population, and context – São Carlos, SP, Brazil, 2024.

*ID	Authors	Country/year	Publication approach	Population	Context
E1	Attrill S, Dymmott A, Wray A.^(22)^	Australia/2019	Quantitative and Qualitative	Clinical supervisors of practice settings.	Universities and health institutions.
E2	Banfield V, Lackie K.^(23)^	Canada/2009	Narrative Literature Review		
E3	Bashatah AS, Al-Ahmary KA, Arifi MA, Asiri YA, Alruthia Y, Metwally AS, et al.^(24)^	Saudi Aarabia/2020	Quasi-Experimental	Teachers in the field of health sciences.	Universities.
E4	Botma Y.^(13)^	South Africa /2019	Qualitative	Experts who are members of the Sub-Saharan Africa Interprofessional Education Network.	*Workgroup of the Sub-Saharan Africa Interprofessional Education Network* (AfrIPEN).
E5	Brashers V, Haizlip J, Owen JA.^(25)^	USA/2019	Mixed methods	Teachers and clinical supervisors from health science practice settings.	Universities.
E6	Christianson TM, Bainbridge L, Halupa C.^(26)^	Canada/2018	Mixed methods	Teachers in the field of health sciences.	Universities.
E7	Coogle LC, Hackett L, Owens MG, Ansello EF, Mathews JH.^(27)^	USA/2016	Quasi-Experimental	Teachers in the field of health sciences.	Universities.
E8	Diggele CV, Roberts C, Burgess A.^(28)^	Australia/2020	Theoretical essay		Universities and health institutions.
E9	Eiff MP, Miller MF, Valenzuela S, Saseen JJ, Zierler B, Carraccio C, et al.^(29)^	USA/2020	Mixed methods	Teachers in the field of health sciences and health professional i with educational actions.	Universities and health institutions.
E10	Fusco NM, Ohtake PJ.^(30)^	USA/2019	Quasi-Experimental	Theachers in Pharmacy courses.	Universities.
E11	Herinek D, Woodward-Kron R, Huber M, Helmer SM, Körner M, et al.^(31)^	Germany/2022	Quantitative	Teachers and clinical supervisors in health science practice settings.	Universities.
E12	Hudak NM, Ross E, Bynum W, Pasha N, Phillips B, Melcher BQ.^(32)^	USA/2021	Mixed methods	Clinical supervisors of practice settings.	Health institutions.
E13	Jacobs JL, Samarasekera DD, Chui WK, Chan SY, Wong LL, Liaw SY, et al.^(33)^	Asia/2013	Qualitative	Teachers in the field of health sciences.	Universities.
E14	Julie H, Hess-April L, Wilkenson J, Cassiem W, Rhoda A.^(34)^	South Africa/2016	Qualitative	Teachers and clinical supervisors in health science practice settings.	Universities and health institutions.
E15	LeGros TA, Amerongen HM, Cooley JH, Schloss EP.^(35)^	USA/2015	Mixed methods	Teachers in the field of health sciences and medical preceptors.	Universities and health institutions.
E16	McCutcheon LRM, Whitcomb K, Craig DC, Klein MS, Burley H, Youngblood T, et al.^(36)^	USA/2017	Quasi-Experimental	Clinical supervisors of practice settings (pharmacy course professionals).	Universities and health institutions.
E17	Mohammed CA, Chaturvedi A, Kamath MG, Ummer SV, Bajaj G.^(37)^	India/2023	Quantitative	Teachers in the field of health sciences. All the participants of the IPE teaching improvement scholarship program.	Universities and health institutions.
E18	Poirier T, Wilhelm M.^(38)^	USA/2014	Qualitative	Teachers in the field of health sciences.	Universities.
E19	Ratka A, Zorek JA, Meyer SM.^(39)^	USA/2017	Narrative Literature Review		
E20	Rider EA, Chou C, Abraham C, Weissmann P, Litzelman DR, Hatem D, et al.^(40)^	USA/2023	Qualitative	Health science faculty members and clinical supervisors from practice settings.	Universities and health institutions.
E21	Schapmire TJ, Head BA, Furman CD, Jones C, Peters B, Shaw MA, et al.^(41)^	USA/2021	Quantitative	Teachers in the field of health sciences.	Universities.
E22	Shelton LR, Thomason AR, Hensarling RW.^(42)^	USA/2022	Quantitative	Teachers in the health sciences field participating in the IPE teaching improvement scholarship program.	Universities and health institutions.
E23	Stokes CK.^(43)^	USA/2021	Narrative Literature Review		
E24	Thistlethwaite JE, Vlasses PH.^(44)^	Canada/2021	Theoretical essay/ Book chapter		
E25	Waters L, Marrs SA, Tompkins CJ, Fix R, Finucane S, Coogle CL, et al.^(45)^	USA/2022	Mixed methods	Teachers in the field of health sciences.	Universities.
E26	Yorke AM, Hoelcher DC, Stalburg CM, Daniels T, Aebersold M, Patterson V, et al.^(46)^	USA/2022	Mixed methods	Teachers in the field of health sciences.	Universities.

Caption: *ID = Study identification.

Source: Prepared by the authors.

**Figure 2:**
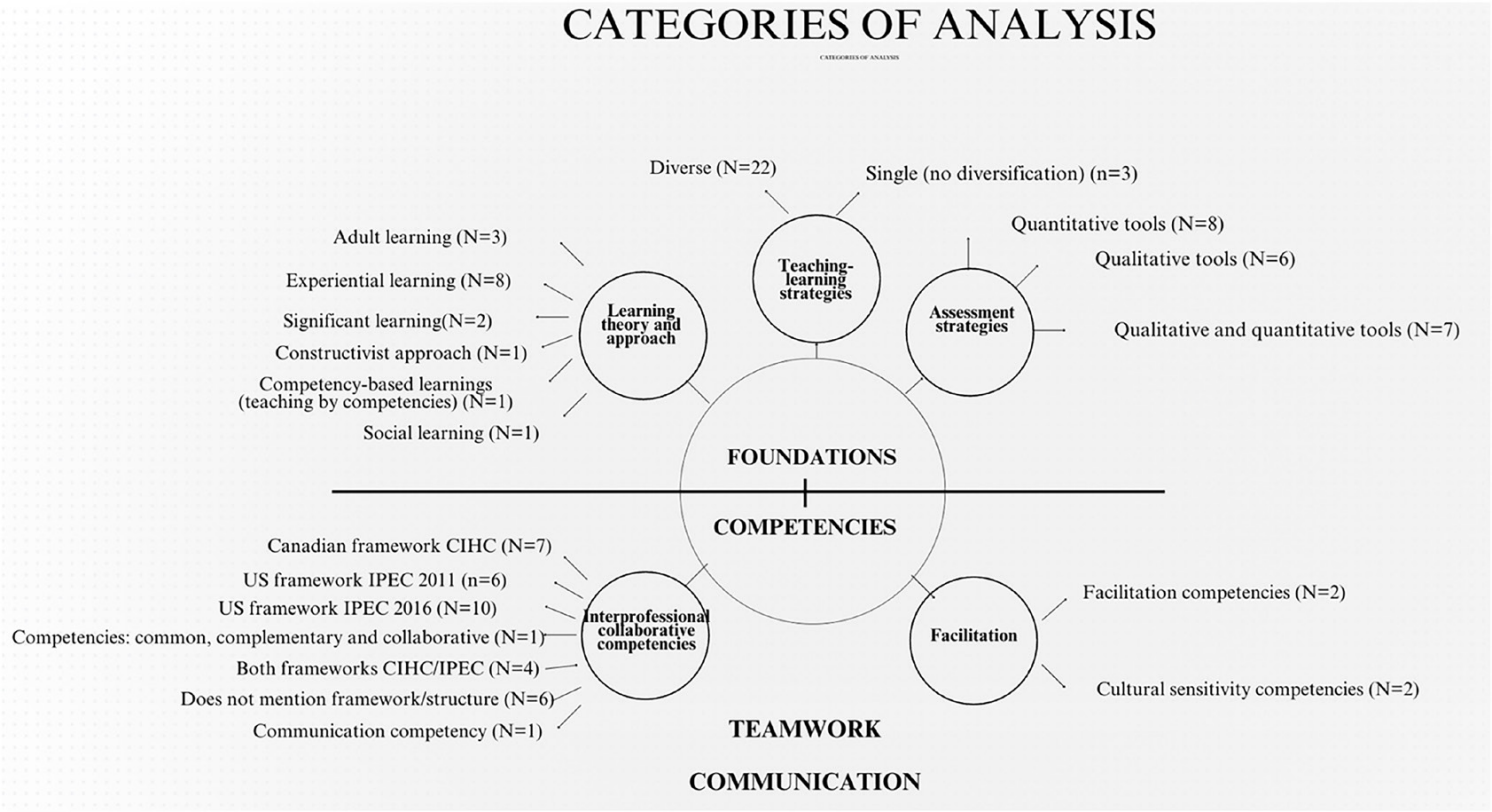
Summary of results in the analysis categories – São Carlos, SP, Brazil, 2024.

**Source:** Figure created in Canva by the authors.

The data analysis revealed two categories of faculty development competencies that contributed to the objective of this scoping review: 1) Interprofessional collaborative and facilitation competencies for IPE; and 2) Pedagogical rationale for IPE actions: theories, learning approaches, teaching-learning strategies, and assessment processes developed by faculty in IPE. The themes that emerged from the data analysis are presented in Chart 3 and summarized in Figure 2.

**Chart 3:** Competencies (concept) and theoretical foundations for faculty development in interprofessional health education – São Carlos, SP, Brazil, 2024.

*ID	Competencies (concept)	Theoretical foundations		
		Theories of learning	Teaching-learning	Evaluation process
E1	Cited, but did not indicate a framework/structure for collaborative skills.	Experiential learning.	Workshops, interactive methods, reflective learning, interprofessional group activities, role-play, feedback, and general communication strategies.	Qualitative instrument: inductive thematic analysis supported by Kirkpatrick’s typology. Quantitative instrument with Likert scale. Application: before and after the teacher development initiative at IPE.
E2	Collaborative, specific and common^(47)^. Skills for facilitation in IPE^(48)^. Cultural sensitivity and safety skills^(49)^.	Experiential learning.	Problem-based learning.	No evidence.
E3	Collaboratives^(15)^.	No evidence.	Workshops, workshops, and small group learning.	Quantitative instrument based on a validated scale^(50)^, Likert scale. Application: before and after teacher development initiative in IPE.
E4	Cited, but did not indicate a framework/structure for collaborative competencies.	Competency-based learning.	No evidence.	No evidence.
E5	Collaboratives^(16)^.	No evidence.	Icebreaker activities, meetings, case studies, simulations, online modules, videos, mentoring workshops, group discussions, appreciative inquiry activities, reflection, and portfolio entries.	Qualitative instruments: feedback, knowledge test, observational checklist, reflection exercises,debriefing, and interviews. Quantitative instruments: questionnaires with a Likert scale. Application: the authors did not report.
E6	Collaboratives^(14)^.	No evidence.	Face-to-face workshops, online activities, small group work, pair reflection exercises, small and large group discussions.	Quantitative instruments: Likert scale and validated questionnaire^(51)^. Application: before and after the teacher development initiative in IPE.
E7	Collaboratives^(15)^.	No evidence.	Seminars and clinical cases. The authors report having used other strategies, but no details were provided.	Quantitative instruments such as self-perception scales. Application: before and after the initiative for teacher development in IPE.
E8	Collaboratives^(14,16)^.	Aprendizagem significativa.	Small group learning, online forums, interprofessional simulations, peer learning, reflection activities, flipped classroom, and summary at the end of the class.	Qualitative instrument: peer feedback. Application: the authors did not report.
E9	Collaboratives^(15)^.	No evidence.	Workshops.	Qualitative instrument: participant observation. Quantitative instrument: questionnaires. Application: before, during, at the end of, and one year after the conclusion of the teacher development initiative at the IPE.
E10	Collaboratives^(16)^.	Experiential learning.	Case study on the topic of communication.	Qualitative instrument: feedback. Validated quantitative instrument^(52)^. Application: before and after teacher development initiative in IPE.
E11	Cited, but did not indicate, a framework/structure for collaborative competencies.	No evidence.	Face-to-face meetings, skills training (no details provided on skills) online lectures, workshops, and discussion facilitated by tutors.	No evidence.
E12	Collaboratives^(16)^.	Experiential and social learning.	Pre-reading, presentations followed by discussions for small and large groups, reflective practice, peer learning, and facilitation skills practice.	Qualitative instrument: semantic thematic analysis. Quantitative instrument: Likert scale questionnaires. Application: before and after the teacher development initiative at IPE.
E13	Collaboratives^(16)^.	No evidence.	Meetings, briefings, lectures, and workshops.	Qualitative instruments: reflection exercises and group reports. Application: did not report when the assessments were applied.
E14	Collaboratives^(14)^.	Experiential learning.	Workshops and small group meetings followed by presentations.	Qualitative instruments: small group discussions and thematic analysis. Application: after the teacher development initiative at IPE.
E15	Collaboratives^(15)^. Facilitation skills in IPE^(23,48)^. Cultural sensitivity and safety skills^(49)^.	Adult learning, experiential and constructivist.	Group discussion, exchange of experiences, and reflection activities.	Qualitative instrument: feedback and debriefing. Quantitative instruments: validated scale^(53)^. Application: before, during, at the end of, and one month after the conclusion of the teacher development initiative at the IPE.
E16	Collaboratives^(15)^.	Experiential learning..]	Structured teaching exercise model based on interprofessional objectives (iOSTE) and lectures.	Qualitative instrument: rubric assessment and debriefing. Quantitative instrument: questionnaires. Application: before and after the teacher development initiative at IPE.
E17	Cited, but did not indicate, a framework/structure for collaborative competencies.	No evidence.	Projet-based learning.	Quantitative instrument validated questionnaire^(54)^. Application: before and after the initiative for teacher development in IPE.
E18	Collaboratives^(15)^.	Experiential learning.	Seminars, posters, thematic roundtable with active learning, case-based simulations, and collaborative discussion groups.	Qualitative instrument: questionnaires. Application: before, on the day of the activity, and at the end of the initiative for teacher development in IPE.
E19	Collaboratives^(14)^.	Experiential learning.	Lectures, workshops, seminars, case studies, role-playing, standardized patients, discussions, posters, interactive plenary sessions, and just-in-time training sessions.	Quantitative instruments: validated scales^(50,55)^. Application: before and after the initiative for teacher development in IPE.
E20	Collaboratives^(14,16)^.	Experiential and significant learning.	Grounding activities at check-in and check-out; small groups, circle, face-to-face with interaction between participants; writing and reading appreciative inquiry narrative reflection with facilitator mediation; reflection based on case studies; Role-play interprofessional approach to practice bad news communication skills and error disclosure and action plan to develop collaborative skills^(47,48)^.	Qualitative instrument: appreciative questionnaire with narrative reflection. Application: after the teacher development initiative at IPE.
E21	Collaboratives^(14,16)^.	Adult learning.	Situational diagnosis to explore the potential for successful dissemination of innovations in the teacher’s home institution, webinars, interactive online modules, training in filling out planning tools, mentoring, feedback, problem-based learning, process reports, and videos.	Quantitative instrument: validated questionnaire^(56)^. Evaluation: before and after the initiative for teacher development in IPE.
E22	Cited, but did not indicate, a framework/structure for collaborative competencies.	No evidence.	Workshop, online activities, and simulation.	Quantitative instrument: Validated scale^(50)^. Assessment: before and after the initiative for teacher development in IPE.
E23	Cited, but did not indicate, a framework/structure for collaborative competencies.	Cited without detail.	Intentional writing/research as a learning strategy to develop new facilitators, IPE curricula/activities, application of active learning principles, guidance, and leadership.	Qualitative instruments: questionnaires to assess behavioral changes based on the Kirkpatrick typology. Application: the authors did not report.
E24	Collaboratives^(14,16)^.	Adult learning.	Interactive methods (no details provided).	No evidence.
E25	Collaboratives^(16)^.	Experiential learning.	Hybrid sessions, small group activities, case studies, and videos.	Qualitative instruments: formative evaluation, debriefing, and focus groups. Quantitative instruments: summative evaluation and questionnaires based on interprofessional collaborative competencies^(16)^. Evaluation: before and after the initiative for IPE teacher development.
E26	Collaboratives^(16)^ and an additional skill: Intercultural humility.	No evidence.	Case study-based activities.	No evidence.

Caption: *ID = Study identification.

Source: Prepared by the authors.

In the first category, the mapped competencies included: interprofessional collaborative and facilitation competencies for IPE. Interprofessional collaborative competencies for IPE were addressed in different ways in publications based on consolidated theoretical frameworks^(14-16,47)^. The Canadian CIHC framework^(14)^ was mentioned in seven studies in this review^(26,28,34,39–41,44)^. The US IPEC framework^(15)^ published in 2011 was used in six included studies^(24,27,29,35,36,38)^, and its 2016 update^(16)^ was present in 10 studies^(25,28,30,32,33,40,44,45,46)^.

Only one study^(23)^ used the three-competence model to guide IPE: 1) Common competencies, a set of general knowledge for all professions; 2) Complementary or specific competencies, which differentiate one profession from another; and 3) Collaborative competencies, between professionals from the same or different professions, including users, families, and communities^(47)^. Some studies applied both frameworks^(14,16)^ to support their studies^(28,40,41,44)^. In addition, six studies reported the relevance of interprofessional collaborative competencies but did not indicate any framework or structure to compose their analyses^(13,22,31,37,42,43)^, and one of them focused on communication^(22)^.

Among the collaborative interprofessional competencies, 16 of the included studies highlighted the domains of teamwork and communication^(14,15,26,28,30,33–36,38–,41,44–46)^ as a relevant part of the interventions performed, but did not delve into the details of expected attributes and performances.

Two publications^(23,35)^ included theoretical references on facilitation in IPE^(48)^ and cultural sensitivity and safety skills in IPE^(49)^, although published prior to the collaborative competency frameworks/structures, they present similar characteristics.

The competencies for facilitating IPE identified^(48)^ were: 1) Commitment to IPE and PIC; 2) Credibility in relation to IPE; 3) Positive role modeling; 4) Deep understanding of interactive learning methods and confidence in their application; 5) Knowledge of group dynamics; 6) Confidence and flexibility to utilize professional differences in groups; and 7) Balance between individual and group needs, as well as willingness to face difficulties.

The competencies of cultural sensitivity and safety in IPE^(49)^ refer to: 1) Making efforts to change one’s worldview; 2) Understanding fundamental cultural issues; 3) Understanding the culture of the professionals with whom one works; 4) Understanding fundamental cultural issues that relate to the health-illness process; 5) Establishing a relationship of trust with users; 6) Providing a welcoming environment in the context of health care; and 7) Negotiating health interventions with the consent of users.

The second category consisted of publications that presented the pedagogical, theoretical-methodological foundations, learning theories or approaches, teaching-learning strategies, and assessment strategies adopted by faculty. Of the 26 publications included, 15 addressed learning theories to guide faculty development initiatives in IPE: experiential learning^(22,23,30,34–36,38-40,45)^, adult learning^(35,41,44)^, meaningful learning^(28,40)^, constructivist approach^(35)^, competency-based learning (competency-based teaching)^(13)^, and social learning^(32)^.

Most studies detailed teaching and learning strategies (Chart 3), an aspect absent in only one publication, which also did not present the instruments related to the evaluation process^(13)^. In addition, 22 publications described different active and interactive teaching-learning methodologies that favor the exchange of experiences between teachers, supervisors, clinical preceptors, and health professionals^(22,24–36,38–45)^. Three studies mentioned only one teaching-learning strategy for the pedagogical offer^(13,37,46)^. 

The studies used a variety of qualitative and quantitative instruments for evaluation, as well as diversifying the periods in which they were applied. Quantitative assessment instruments were used in eight studies, all with two application moments, before and after the initiatives for the development of IPE^(24,26,27,30,37,39,41,42)^, including *Likert* scales and validated questionnaires, such as *The Interdisciplinary Education Perception Scale* ^(50)^, *The Interprofessional Education for Collaborative Patient-Centred Care Questionnaire* ^(51)^, *Interprofessional Collaborative Competencies Attainment Survey* ^(52)^, *Interprofessional Facilitation Scale* ^(53)^, *Perception of Interprofessional Collaboration Model Questionnaire* ^(54)^ *, Readiness for Interprofessional Learning Scale* ^(55)^, and *Core Competencies for Interprofessional Practice Individual Competency Assessment Tool* ^(56)^.

The qualitative assessment instruments were questionnaires, interviews, reflection exercises, group reports, rubric assessment, small interprofessional group discussions, face-to-face feedback, and self-assessment. Six studies applied qualitative instruments to evaluate initiatives for faculty development in IPE alone^(28,33,34,38,40,43)^. Of the studies analyzed, three did not report when the evaluation instruments were applied^(28,33,43)^ and two applied them only after the initiative^(34,40)^. One study that applied the evaluation instrument only at the end did not detail the learning objectives; however, participants reported positive reactions to the intervention, changes in attitudes and perceptions, and the acquisition of knowledge and skills in a collaborative manner^(34)^.

In total, seven studies applied instruments to evaluate qualitative and quantitative approaches^(22,25,29,32,35,36,45)^. Of these, one did not describe when it was applied^(25)^, two applied it at four different times: before, during, at the end, and some time after the IPE intervention^(29,35)^, and one applied the assessment one year after the intervention, although its benefits were not evaluated^(29)^. The other studies applied instruments at two different times, before and after the intervention^(22,32,36,45)^.

## DISCUSSION

This scoping review allowed us to map the interprofessional collaborative competencies that faculty, teachers, supervisors, clinical preceptors, health professionals, and educators understand to be necessary during the interprofessional training process. The pedagogical and theoretical-methodological foundations covered by the theories, learning approaches, teaching-learning strategies, and assessment strategies were also identified, which consist of enabling principles for competencies for faculty development in IPE. We chose to systematize the aforementioned pedagogical foundations, considering that the field of IPE has specificities that require the application of active, interactive, and shared teaching-learning and assessment strategies. Thus, the findings can contribute to equipping faculty in the process of planning, implementing, and evaluating IPE curriculum proposals and initiatives.

It should be noted that most of the studies identified use the CIHC and IPEC frameworks, demonstrating that their initiatives are aligned with the PIC frameworks/structures^(14–16)^. This review strongly identified the domains of communication and teamwork, which express the intention to strengthen the health workforce from an interprofessional perspective. However, it is important to note that the IPE commitment also highlights user-, family-, and community-centered care, which is present in these analytical frameworks but still incipient in the studies analyzed.

Regarding the heterogeneity of training in IPE faculty development initiatives, the particularities and characteristics of these professionals should be considered, such as part-time or full-time work, teaching qualifications and responsibilities, conditions that may influence the approach and methods of facilitation in IPE^(35)^. In addition, faculty and other facilitators may have difficulty identifying learning opportunities in IPE in practice settings, given their different repertoires and previous experiences^(57)^. In this regard, a study highlighted that supervisors and clinical preceptors identified opportunities for PIC, but teachers did not have the same perception and needed to work together with these agents in practice settings to identify and expand training opportunities^(34)^.

The literature suggests strategies to deal with these difficulties in faculty perception of the real opportunities of IPE. The appointment of agents experienced in IPE facilitation can provide opportunities to experiment with different teaching-learning methods and teamwork dynamics^(35)^. The involvement of agents experienced in IPE can contribute to the dissemination of its pedagogical foundations and assumptions, the valorization of different interprofessional knowledge, and the strengthening of partnerships in teaching-service-community integration, aimed at building experiences and reflections with an emphasis on IPE, from the beginning of the training course.

It was possible to identify and synthesize theories, learning approaches, teaching-learning strategies, and assessment strategies consistent with IPE. As also highlighted in the results of this review, a study revealed that many pedagogical interventions in IPE did not provide precise details on the learning theory chosen to guide the interventions^(58)^. This may be due to a lack of understanding of the learning theories employed in IPE initiatives, but it may also be associated with a lack of pedagogical guidelines, objectives, learning outcomes, and analysis of the impact of IPE initiatives on the teaching staff^(59,60)^. The absence of these theories or pedagogical approaches can hinder the construction and implementation of successful faculty development initiatives in IPE.

The learning theories selected in the studies were applied independently or in combination. Three studies used more than one learning theory to inform pedagogical practices, but did not describe how they were linked to other pedagogical components and their impacts^(32,35,40)^. Learning theories are partial and restricted approaches to specific aspects and areas of learning, so they hardly constitute a complete set of knowledge that elucidates the meaning of the complex phenomena that occur during learning^(61)^. If a theory or approach is not sufficient to understand the complex phenomena of learning, it can be inferred that pedagogical planning should be based on more than one theory or approach so that it can meet the diverse needs of students during IPE activities.

Adult learning theory emphasizes responsibility and protagonism in one’s own learning, which in the case of IPE is also shared between individuals and groups. The principles of adult education emphasize cooperative, collaborative, reflective, and socially constructed learning among people^(62)^, consistent with the principles and values of IPE, which include collaboration, interaction, mutual respect, partnerships, and accountability^(63)^.

Experiences in faculty development in IPE have also revealed a constructivist approach, in which human beings are seen as individuals who are transformed through relationships and interactions with the social and cultural environment in which they live. In other words, learning and transformation through social interactions^(64)^. The constructivist approach can be the basis for the development, delivery, and evaluation of faculty development initiatives in IPE, given that students and health professionals need to interact constantly. Thus, it is necessary to prioritize and encourage learning experiences that promote interprofessional interactions among professionals, students^(35,44)^, and between them and users, families, and communities.

Another perspective presented in the results was experiential learning, in which knowledge is constructed and modified through experiences. Thus, knowledge is not considered an immutable element, as in the traditional approach. Experience results from the interaction between the external and internal worlds of the individual, based on situations that allow the creation and recreation of knowledge^(65)^. During experiences, in the construction and reconstruction of new knowledge, conflicts may arise that result in improved perceptions, understanding, and the acquisition of new skills. In this process, participants in the learning community work together to understand phenomena, make decisions, and solve problems^(66)^, aspects that reinforce the need for IPE experiences in real health practice settings.

The publications analyzed also dealt with active and interactive approaches, given the importance of intentional exchange between students, educators, supervisors, preceptors, and teachers^(44,59)^, which are aligned with the principles and values of IPE. The active and interactive methodology expands the possibility of critical-reflective teaching and learning, student commitment and protagonism, opportunities to improve collaborative skills^(63)^, and make learning more attractive and effective^(58)^.

In short, most studies mentioned the interprofessional collaborative skills provided by the experiential approach, among other theories and pedagogical foundations, either in isolation or in combination, such as adult learning, meaningful learning, and social constructivism. The common link between these approaches is the shift of protagonism in learning from the faculty to the student.

The literature recommends that professional health training experiences present the theories that underpin them in a more explicit and robust manner, the objective of education, the nature of knowledge, what is valued and included in the curriculum, the meaning of learning and how it is assessed, the roles of teachers and students in the process, i.e., that there is alignment between teaching, learning, and assessment practices and paradigmatic values and assumptions^(67)^. There is also research indicating progress in the theorization of teaching-learning practices imbued with emotions, with recognition that education is beyond cognitivism, an affective, social, and political process^(68)^. Another study highlighted the difficulty of faculty presenting the theory underlying their experiences in advance, indicating it only a posteriori^(69)^. In a review of IPE interventions, a set of recommendations was indicated for educators to design, implement, and evaluate IPE to achieve interprofessional competencies. Specifically in relation to faculty development, the focus of this review, they identified that teachers receive training before becoming involved in the intervention, but there is no information on whether this training is specific to facilitating IPE or of a general pedagogical nature^(70)^.

The above studies^(67–70)^ reinforce the findings of this review, weaknesses between theoretical principles and teaching practice, empirical experiences to the detriment of deeper theoretical reflection, lack of consensus on which theories are at the origin of IPE and PIC and how to apply them. The difficulties listed express the complexity of constructing IPE training experiences, reasons that may also be related to the tenuous presence of theories and theoretical foundations in IPE faculty development initiatives.

Regarding the evaluation process, the Center for the Advancement of Interprofessional Education (CAIPE) proposes the implementation of dynamic, diversified, and continuous strategies throughout the IPE action^(63)^. The evaluation process should be considered in the project design, defining the instruments used, aligned with the objectives, learning outcomes, and moments of their application^(71)^.

With regard to the articulation of IPE to strengthen health system practices, which in Brazil refers to the SUS, although no studies have been conducted in the Brazilian health system, evidence suggests that higher education institutions (HEIs) are responsible for this through faculty development policies and programs aimed at improving health outcomes, from the perspective of the interdependence between the health and education systems. The social commitment of HEIs lies in training a workforce prepared to meet the needs of users and health systems, with an emphasis on quality. Prioritizing teachers and facilitators in the development of interprofessional collaborative skills enables the strengthening of teamwork, interprofessional collaboration, and user-centered care.

Despite the challenge of training in interprofessional collaborative competencies integrated with the needs of individuals, families, and communities, and the recognition of the importance of moving in this direction, courses that do not include periods in the curriculum for IPE still predominate in Brazil, and when they do occur, they are sporadic activities from a multiprofessional perspective^(72)^.

Faculty training for IPE has the potential to stimulate recognition of opportunities for interprofessional training in practice contexts, of relevant opportunities that impact knowledge, skills, and confidence in the teaching-learning process^(73)^, whose starting point is the occurrence of more flexible and articulated curricula.

Although the study complied with the methodological procedures recommended for a scoping review, with extensive mapping of the literature and no time restrictions, it is important to consider its limitations. Restricting the search to three languages, Portuguese, English, and Spanish, may have led to losses, especially of publications in other languages. Only one publication identified as gray literature was included in the findings, a result that may be due to inaccuracies in mapping this type of publication. The heterogeneity of the designs of the publications included for the construction of syntheses and the lack of evaluation of the methodological quality of the included studies, a step that is rarely present in scoping reviews.

## CONCLUSION

This scoping review aimed to map faculty development competencies for IPE, which required the analysis of interprofessional collaborative and facilitation competencies present in educational interventions, with an emphasis on the domains of communication and teamwork.

The evidence produced highlights the need for future studies on the development of IPE teachers and facilitators with an emphasis on teaching-service-community integration, as well as on the construction, dissemination, and application of a framework of competencies from a pedagogical perspective in IPE. Advances are also needed in research demonstrating the links between pedagogical theories/approaches and competencies for faculty development in IPE, as well as the results of evaluations after initiatives, especially in the long term.

This scoping review concludes that the domains of competencies for faculty development in IPE can be divided into two groups: 1) Interprofessional collaborative and facilitation competencies for IPE and 2) Pedagogical, theoretical, and methodological foundations for faculty development in IPE: theories, learning approaches, teaching-learning strategies, and assessment. This mapping contributes to the theoretical foundation for planning, implementing, and evaluating initiatives for the development of IPE and its agents, teachers, clinical supervisors, preceptors in practice settings, health professionals, and students themselves, in order to strengthen interprofessionalism.

Based on the findings, it is recommended that health course managers, faculty development program coordinators, and educational policy makers recognize the importance of learning theories in strengthening IPE and faculty development, with the recommendation that these pedagogical foundations/theories be made explicit and aligned and consistent with the characteristics of IPE. Furthermore, investment in training spaces is recommended, with broad participation of the different agents jointly responsible for health training, in addition to the teaching staff.

## DATA AVAILABILITY

All datasets supporting the findings of this study are available upon request from the corresponding author [Jaqueline Alcântara Marcelino da Silva].

## Footnotes

1 In this publication, faculty are considered to be those who plan, implement, and evaluate curricular actions in health education, and facilitators are considered to be other agents involved in health education, such as preceptors, supervisors, tutors, and mentors, who do not always participate in all stages of the educational processes.
