# Supplementary material for: Competencies and pedagogical foundations for faculty development in interprofessional health education: a scoping review
Source: Rev Esc Enferm USP. 2025 Nov 3;59:e20240421. doi: 10.1590/1980-220X-REEUSP-2024-0421en (PMC12594216; doi:10.1590/1980-220X-REEUSP-2024-0421en)
Supplement: Translation

# Translation: Competências e fundamentos pedagógicos para o desenvolvimento docente na educação interprofissional em saúde: revisão de escopo*

**DOI:** 10.1590/1980-220X-REEUSP-2024-0421pt

**Authors:** Daniella Rosaly Leite, Patrícia Silva Carneiro, Vivian Aline Mininel, Rosana Aparecida Salvador Rossit, Valéria Marli Leonello, Geisa Colebrusco de Souza Gonçalves, Jaqueline Alcântara Marcelino da Silva

**Affiliations:** 1 Universidade Federal de São Carlos, São Carlos, SP, Brasil.; 2 Universidade Federal de Goiás, Goiânia, GO, Brasil.; 3 Universidade Federal de São Paulo, São Paulo SP, Brasil.; 4 Universidade de São Paulo, São Paulo SP, Brasil.

**Keywords:** Competência Profissional, Educação Baseada em Competências, Capacitação de Professores, Docentes, Educação Interprofissional

## Abstract

**Objetivo:**

Mapear as competências para o desenvolvimento docente na educação interprofissional em saúde.

**Método:**

Trata-se de uma revisão de escopo, baseada nas recomendações do Instituto Joanna Briggs com a questão norteadora: “Quais competências são utilizadas para o desenvolvimento docente na educação interprofissional?” As buscas foram realizadas nas bases de dados MEDLINE/OVID, ERIC, CINAHL, Scopus, Web of Science, Google Scholar, EBSCO Open Dissertations, BDTD, Teses e Dissertações da CAPES, OpenGrey e MedNar, sem recorte temporal e nos idiomas português, espanhol e inglês.

**Resultados:**

Foram incluídas 26 publicações, agrupadas em duas categorias: a primeira refere-se às competências colaborativas interprofissionais e de facilitação para a educação interprofissional, com destaque para os domínios da comunicação e do trabalho em equipe. A segunda diz respeito à fundamentação pedagógica da EIP, teorias, abordagens, estratégias de ensino-aprendizagem e de avaliação, nas ações de desenvolvimento docente para educação interprofissional.

**Conclusão:**

As evidências mapeadas contribuem para fortalecer as iniciativas de desenvolvimento docente e para o avanço da fundamentação teórica e metodológica da educação interprofissional em saúde.

## INTRODUÇÃO

As organizações de saúde têm lidado com desafios ocasionados pela fragmentação dos sistemas de saúde e das práticas de cuidado que comprometem a disponibilidade, acesso, qualidade dos serviços, e as dificuldades inerentes à formação de profissionais de saúde. Existe uma premência por estratégias inovadoras, com ênfase na elaboração de políticas e programas que consolidem a formação e o trabalho na área da saúde^(1,2)^, visto que os serviços de saúde requerem profissionais preparados a partir de competências colaborativas interprofissionais para lidar com as mudanças e os desafios do cuidado à saúde^(2)^.

A educação interprofissional em saúde (EIP) busca contribuir para a reformulação das matrizes curriculares dos cursos de graduação e de pós-graduação, com o objetivo de preparar os profissionais para trabalhar em equipe de forma eficiente, com foco na interação, integração e colaboração^(3)^, cujo cuidado seja centrado na pessoa, no grupo ou na população^(4)^. A EIP é um movimento global, e no contexto brasileiro, encontra possibilidade de ocorrência nos serviços do Sistema Único de Saúde (SUS), em espaços formativos diferentes: graduação, residências e educação permanente em saúde. Nos referidos contextos formativos, a EIP contribui para mobilizar reflexões sobre o processo de trabalho e a coprodução voltada ao fortalecimento das competências colaborativas interprofissionais e de práticas comprometidas com as necessidades de saúde dos usuários e da população, como protagonistas do cuidado^(5)^.

Com a expansão da EIP, torna-se crucial preparar os docentes para lidarem com os desafios dessa abordagem educacional inovadora^(2,6)^. Os docentes são requisitados para liderar, facilitar e elaborar currículos de EIP, porém, apresentam dificuldades para assumir essas atribuições e responsabilidades^(7)^, por não terem sido expostos às atividades de aprendizagem e práticas interprofissionais em seu processo de formação profissional ou de prática docente^(8)^, com escassas iniciativas de desenvolvimento docente, sobretudo na perspectiva da EIP.

Apesar de o termo docente ser amplamente utilizado para professores universitários, esses não são os únicos agentes envolvidos na formação em saúde. Amplia-se a compreensão de que profissionais que atuam nos serviços na rede de atenção devem também participar de formação para desenvolvimento de ações em EIP, com reconhecimento de sua contribuição na formação profissional^(9)^. Desta forma, reconhece-se que nas iniciativas de EIP estão envolvidos facilitadores que são profissionais da saúde, doutores, mestres, especialistas em formação, preceptores, tutores e supervisores, que atuam como agentes corresponsáveis pela formação em saúde e necessitam de oportunidades formativas para atuar no ensino em saúde.

Estudo de revisão sistemática indicou a necessidade de adotar iniciativas para o desenvolvimento docente na EIP, uma vez que dentre os principais aspectos para uma intervenção interprofissional bem-sucedida estão a capacidade da facilitação dos docentes, a compreensão da abordagem educacional, das estratégias de ensino-aprendizagem, dos processos avaliativos e das competências na EIP^(10)^. Na mesma direção, um estudo apontou que a prática da EIP enfatizou as competências colaborativas interprofissionais para os estudantes, porém, as competências docentes de facilitação foram negligenciadas^(11)^.

Assim, o êxito de uma intervenção educativa requer objetivos de aprendizagem, resultados esperados, conteúdos, estratégias de ensino-aprendizagem e avaliação, além das competências a serem desenvolvidas, encadeadas de forma coerente^(12)^. Para que o planejamento, implementação e avaliação dos currículos sejam ancorados na EIP, os docentes precisam de oportunidades para desenvolver novas competências^(7,10,13)^.

Frente ao exposto e diante da ausência de estudos prévios, identificou-se a necessidade de produzir o mapeamento das competências para o desenvolvimento docente na EIP, com a intenção de minimizar as lacunas encontradas na literatura, direcionar políticas e programas no contexto local (institucional) e no macro contexto da formação em saúde (sistema), para subsidiar futuras iniciativas na temática. O objetivo desta revisão de escopo foi mapear as competências para o desenvolvimento docente na EIP.

## REFERENCIAL TEÓRICO

Na literatura, destacam-se dois quadros que descrevem competências colaborativas interprofissionais, um canadense^(14)^ e um estadunidense^(15)^, embora não abordem o desenvolvimento docente, indicam competências para os profissionais que atuam nos sistemas de saúde. O quadro do *Canadian Interprofessional Health Collaborative* (CIHC)^(14)^ engloba quatro competências: clareza de papéis; funcionamento de equipe; resolução de conflitos interprofissionais; e, liderança colaborativa, dependentes de dois domínios de competências principais: comunicação interprofissional e atenção centrada nos usuários, famílias e comunidades.

O quadro canadense visa identificar conhecimentos, habilidades, atitudes, comportamentos e julgamentos necessários para atingir a excelência e aplicá-los em situações de colaboração interprofissional. Desse modo, tais competências podem contribuir no planejamento das ações de EIP, elaboradas por docentes e demais facilitadores^1^, visando ações educativas bem-sucedidas^(14)^.

Em 2024 o quadro CIHC foi atualizado, com mudanças nas terminologias destinadas a aprimorar a colaboração interprofissional. Dentre as alterações, destaca-se a inclusão de parceiros dos cuidados e de serviços de saúde, bem como de todas as pessoas envolvidas nas relações de cuidado. O objetivo da atualização foi melhorar os cuidados de saúde ofertados, por meio de parcerias colaborativas e centradas no relacionamento, além de facilitar a tomada de decisões conjuntas sobre as necessidades sociais e de saúde. Na base do quadro foram apresentados os fatores que influenciam a aplicação das competências, tais como a inclusão, o acesso e a equidade, relacionados à complexidade e ao contexto dos cuidados e serviços de saúde^(4)^.

O quadro estadunidense, *Interprofessional Education Collaborative* (IPEC)^(15)^ apresenta competências essenciais de prática colaborativa. Publicado em 2011, com quatro áreas temáticas relacionadas a EIP: valores e ética; funções e responsabilidades; comunicação interprofissional; equipe e trabalho em equipe^(15)^, foi atualizado em 2016, com destaque da alteração de apresentar as quatro áreas temáticas citadas num único domínio: competência colaborativa interprofissional, com ênfase da centralidade do cuidado nos usuários, famílias e comunidades^(16)^.

A última versão do quadro IPEC publicada em 2023^(17)^ expressa a prática interprofissional colaborativa (PIC) como essencial para alcançar melhores resultados em saúde, na perspectiva do cuidado seguro, de qualidade, acessível, equitativo e centrado na pessoa. Houve manutenção do domínio singular da competência colaboração interprofissional e das quatro áreas (valores e ética; papéis e responsabilidades; comunicação; equipes e trabalho em equipe) com a indicação do compromisso da formação na saúde ao longo da vida, visando que recém-formados e experientes profissionais de saúde se engajem na construção das referidas competências.

## MÉTODO

### Tipo do Estudo

Trata-se de uma revisão de escopo (*scoping review*) pautada nas recomendações do Instituto Joanna Briggs (JBI) e no *Preferred Reporting Items for Systematic Reviews and Meta-Analysis Extension for Scoping Reviews* (PRISMA) - *Extension for Scoping Reviews* (PRISMA-ScR): *Checklist and Explanation* ^(18)^. A revisão de escopo tem como objetivo mapear os principais conceitos que apoiam determinada área do saber, examinar a extensão, o alcance do objeto da investigação, substanciar e divulgar os dados, bem como identificar lacunas existentes^(19)^. O protocolo foi elaborado e revisado pelos autores e registrado prospectivamente na plataforma *Open Science Framework* (OSF): https://osf.io/6gazx/. O estudo foi conduzido em etapas sequenciais: identificação da questão da pesquisa, extração, análise e apresentação dos resultados^(19)^. Durante o processo, os autores procederam a discussões para alinhamentos e consensos.

### Questão de Pesquisa

A estratégia mnemônica População, Conceito e Contexto (PCC) foi utilizada para a construção da pergunta de pesquisa. Esta revisão teve como população (P) docentes, preceptores, tutores, mentores, professores ou facilitadores do ensino superior dos cursos de ciências da saúde, o conceito (C), competências relacionadas ao desenvolvimento docente para a EIP e o contexto (C) universidades e/ou cenários de práticas da formação em saúde. Dessa forma, adotou-se a seguinte questão norteadora: “Quais competências são utilizadas para o desenvolvimento docente na EIP?”

### Critérios de Elegibilidade

Foram incluídos estudos teóricos (ensaios teóricos e revisões de literatura), estudos empíricos primários e secundários, quantitativos, qualitativos mistos e quase-experimentais publicados nos idiomas português, espanhol e inglês, sem restrição de tempo, com população composta por docentes do ensino superior da área de ciências da saúde e preceptores de campo que abordassem as competências necessárias para o desenvolvimento docente no contexto da EIP. Portanto, consideramos nesta revisão, além de docentes todos os profissionais da saúde que atuam na formação dos estudantes (doutores, mestres, especialistas, bacharéis), preceptores, supervisores e facilitadores que participam da formação em saúde. A literatura cinza, como dissertações, teses, livros e/ou capítulos de livros e manuais foi incluída quando respondeu à questão da pesquisa. Os critérios de exclusão contemplaram resenhas, editoriais, anais de congressos e eventos.

### Fontes de Dados e Estratégia de Busca

A estratégia de busca foi construída pelos autores do estudo com apoio de bibliotecária experiente, a partir de uma combinação de Descritores em Ciências da Saúde (DeCS), Medical Subject Headings (MeSH), e operadores booleanos AND e OR, apresentados no Quadro 1. As buscas ocorreram em dezembro de 2023, nas seguintes bases de dados: *Medical Literature Analysis and Retrieval System* (MEDLINE/OVID), *Education Resources Information Center* (ERIC), *Cumulative Index to Nursing and Allied Health Literature* (CINAHL) via EBSCO, *Web of Science Institute for Scientific Information,* Scopus *SciVerse*, *Google Scholar*, EBSCO *Open Dissertations*, Catálogo de Teses e Dissertações (CTD) da Coordenação de Aperfeiçoamento de Pessoal de Nível Superior (CAPES), Biblioteca Digital Brasileira de Teses e Dissertações (BDTD), *Deep Web Search Engine* (MedNar) e *System for Information on Grey Literature in Europe* (OpenGrey). Para identificar outros estudos elegíveis foram realizadas buscas manuais nas referências dos estudos selecionados.

**Quadro 1:** Estratégia de busca nas bases de dados – São Carlos, SP, Brasil, 2024.

Base	Expressão
MEDLINE/OVID	**S1** (Faculty* OR Mentor* OR Professor* OR Tutor* OR Teacher* OR Preceptor* OR Instructor* OR Facilitat* OR Development).mp. [mp = title, abstract, original title, name of substance word, subject heading word, floating sub-heading word, keyword heading word, organism supplementary concept word, protocol supplementary concept word, rare disease supplementary concept word, unique identifier, synonyms] **S2** (Competency Based Education OR Competency Based Education OR Education, Competency-Based OR Competency-Based Educations OR Education, Competency Based OR Educations, Competency-Based OR Competency).mp. [mp = title, abstract, original title, name of substance word, subject heading word, floating sub-heading word, keyword heading word, organism supplementary concept word, protocol supplementary concept word, rare disease supplementary concept word, unique identifier, synonyms] **S3** (Interprofessional education OR interprofessional education OR IPE OR multi-professional education OR multi-professional education OR multi-professional education OR multidisciplinary education OR multi-disciplinary education).mp. [mp = title, abstract, original title, name of substance word, subject heading word, floating sub-heading word, keyword heading word, organism supplementary concept word, protocol supplementary concept word, rare disease supplementary concept word, unique identifier, synonyms] **S4** (1 and 2 and 3) ((Faculty* OR Mentor* OR Professor* OR Tutor* OR Teacher* OR Preceptor* OR Instructor* OR Facilitat* OR Development) AND (Competency Based Education OR Competency Based Education OR Education, Competency-Based OR Competency-Based Educations OR Education, Competency Based or Educations, Competency-Based OR Competency) AND (Interprofessional education OR interprofessional education OR IPE OR multi-professional education OR multi-professional education OR multi-professional education or multidisciplinary education OR multi-disciplinary education)).mp. [mp = title, book title, abstract, original title, name of substance word, subject heading word, floating sub-heading word, keyword heading word, organism supplementary concept word, protocol supplementary concept word, rare disease supplementary concept word, unique identifier, synonyms, population supplementary concept word, anatomy supplementary concept word]
ERIC e CINAHL	**S1** (Faculty* OR Mentor* OR Professor* OR Tutor* OR Teacher* OR Preceptor* OR Instructor* OR Facilitat* OR Development) AND (Competency Based Education OR Competency Based Education OR Education, Competency-Based OR Competency-Based Educations OR Education, Competency Based OR Educations, Competency-Based OR Competency) AND (Interprofessional education OR interprofessional education OR IPE OR multi-professional education OR multi-professional education OR multi-professional education OR multidisciplinary education OR multi-disciplinary education)
Web of Science	**S1** (Faculty* OR Mentor* OR Professor* OR Tutor* OR Teacher* OR Preceptor* OR Instructor* OR Facilitat* OR Development) AND (“Competency-Based Education” OR “Competency Based Education” OR “Education, Competency-Based” OR “Competency-Based Educations” OR “Education, Competency Based” OR “Educations, Competency-Based” OR Competency) AND (“Interprofessional education” OR “interprofessional education” OR IPE OR “multiprofessional education” OR “multi-professional education” OR “multiprofessional education” OR “multidisciplinary education” OR “multi-disciplinary education”)
Scopus	**S1** TITLE-ABS-KEY Faculty* OR Mentor* OR Professor* OR Tutor* OR Teacher* OR Preceptor* OR Instructor* OR Facilitat* OR Development) AND (Competency Based Education OR Competency Based Education OR Education, Competency-Based OR Competency-Based Educations OR Education, Competency Based OR Educations, Competency-Based OR Competency) AND (Interprofessional education OR interprofessional education OR IPE OR multi-professional education OR multi-professional education OR multi-professional education OR multidisciplinary education OR multi-disciplinary education)
Google Scholar	**S1** (Faculty* OR Mentor* OR Professor* OR Tutor* OR Teacher* OR Preceptor* OR Instructor* OR Facilitat* OR Development) AND (Competency Based Education OR Competency Based Education OR Education, Competency-Based OR Competency-Based Educations OR Education, Competency Based OR Educations, Competency-Based OR Competency) AND (Interprofessional education OR interprofessional education OR IPE OR multi-professional education OR multi-professional education OR multi-professional education OR multidisciplinary education OR multi-disciplinary education)
EBSCO *Open Dissertations*	**S1** (Faculty* OR Mentor* OR Professor* OR Tutor* OR Teacher* OR Preceptor* OR Instructor* OR Facilitat* OR Development) AND (Competency Based-Education OR Competency Based Education OR Education, Competency-Based OR Competency-Based Educations OR Education, Competency Based OR Educations, Competency-Based OR Competency) AND (Interprofessional education OR interprofessional education OR IPE OR multi-professional education OR multi-professional education OR multi-professional education OR multidisciplinary education OR multi-disciplinary education)
BDTD/Teses e dissertações da CAPES	**S1** “Professor AND Competências AND Educação Interprofissional”
MedNar	**S1** “Faculty AND Competency AND Interprofessional Education Development”
OpenGrey	**S1** “Faculty AND Interprofessional”

Fonte: Elaborado pelos autores.

### Seleção dos Estudos

Os resultados obtidos foram exportados para o *software Rayyan®* ^(20)^, para proceder a remoção automática dos estudos duplicados. A triagem e a seleção dos estudos foram realizadas mediante procedimento de duplo cego, por dois revisores independentes, em duas etapas, a partir da seleção por títulos e resumos, na primeira etapa, e por leitura dos textos completos, na segunda. As divergências na seleção foram resolvidas por terceiro revisor. As listas de referências dos estudos selecionados na segunda etapa também passaram pela análise do terceiro revisor para confirmar a pertinência de inclusão dos estudos sobre a temática. Os resultados das buscas e os estudos selecionados estão detalhados no fluxograma *Preferred Reporting Items for Systematic Reviews and Meta-Analyses Extension for Scoping Reviews* (PRISMA-ScR)^(18)^, apresentado a seguir:

### Análise dos Dados e Síntese dos Resultados

Para extração dos dados dos estudos selecionados foi elaborada uma planilha no *software Microsoft Excel®*. Selecionaram-se as seguintes informações relevantes dos estudos: 1) Caracterização: autoria, país, ano, abordagem da publicação, população e contexto; 2) Identificação do estudo, competências colaborativas interprofissionais, teorias ou abordagens de ensino- aprendizagem e processo-avaliativo.

Os dados foram analisados por meio de análise de conteúdo temática descritiva e dedutiva^(19)^, que consistiu na organização das informações em três etapas: pré-análise; exploração do material; e interpretação dos resultados^(21)^, correspondente às três etapas de análise de conteúdo em revisões de escopo: preparação, organização e elaboração de relatórios^(19)^. Assim, o material selecionado foi organizado e codificado em unidades de registro (temas) e indicador não frequencial, seguido pela categorização, classificação dos temas por diferenciação e reagrupamento^(21)^, a análise dedutiva baseou-se nos construtos teóricos da EIP e nos quadros de competências do CIHC^(14)^ e IPEC^(15-17)^.

## RESULTADOS

As buscas totalizaram 1.776 registros, 26 estudos atenderam os critérios de elegibilidade e foram incluídos nesta revisão conforme demostrado na Figura 1. Foram publicados entre os anos de 2009 e 2023, provenientes dos seguintes países: Estados Unidos (n = 15; 57%), Canadá (n = 3; 11%), África do Sul (n = 2; 8%), Austrália (n = 2; 8%), Alemanha (n = 1; 4%), Arábia Saudita (n = 1; 4%), Ásia (n = 1; 4%) e Índia (n = 1; 4%). A maioria eram pesquisas de método misto (n = 8; 31%), qualitativas (n = 5; 19%), quantitativas (n = 4; 15%), quase-experimental (n = 4; 15%), revisão de literatura narrativa (n = 3; 12%) e ensaio teórico (n = 2; 8%).

**Figura 1:** Fluxograma PRISMA- ScR, identificação, triagem e seleção dos estudos incluídos na revisão de escopo – São Carlos, SP, Brasil, 2024.

**Fonte:** Elaborado pelos autores.

Em relação ao contexto dos estudos, 11 (42%) foram conduzidos em instituições de ensino superior (IES), 10 (39%) em instituições de saúde e IES, um (4%) na Rede de Educação Interprofissional da África do Sul e quatro (15%) não tinham contexto definido, por serem ensaios teóricos ou revisões narrativas.

Como participantes, os estudos incluíram docentes, supervisores clínicos e preceptores dos cenários de prática, profissionais de saúde envolvidos na formação e especialistas de interprofissionalidade, atuantes na área de ciências em saúde. Os dados extraídos das publicações estão apresentados no Quadro 2.

**Quadro 2:** Caracterização dos estudos segundo autor, país, ano de publicação, abordagem da publicação, população e contexto – São Carlos, SP, Brasil, 2024.

*ID	Autoria	País/Ano	Abordagem da publicação	População	Contexto
E1	Attrill S, Dymmott A, Wray A.^(22)^	Austrália/2019	Quantitativo e Qualitativo	Supervisores clínicos dos cenários de prática.	Universidades e instituições de saúde.
E2	Banfield V, Lackie K.^(23)^	Canadá/2009	Revisão da Literatura Narrativa		
E3	Bashatah AS, Al-Ahmary KA, Arifi MA, Asiri YA, Alruthia Y, Metwally AS, et al.^(24)^	Arábia Saudita/2020	Quase-Experimental	Docentes da área de ciências da saúde.	Universidades.
E4	Botma Y.^(13)^	África do Sul/2019	Qualitativo	Especialistas membros da Rede de Educação Interprofissional da África Subsaariana.	*Workgroup of the Sub-Saharan Africa Interprofessional Education Network* (AfrIPEN).
E5	Brashers V, Haizlip J, Owen JA.^(25)^	EUA/2019	Métodos Mistos	Docentes e Supervisores clínicos dos cenários de prática da área de ciências da saúde.	Universidades.
E6	Christianson TM, Bainbridge L, Halupa C.^(26)^	Canadá/2018	Métodos Mistos	Docentes da área de ciências da saúde.	Universidades.
E7	Coogle LC, Hackett L, Owens MG, Ansello EF, Mathews JH.^(27)^	EUA/2016	Quase-Experimental	Docentes da área de ciências da saúde.	Universidades.
E8	Diggele CV, Roberts C, Burgess A.^(28)^	Austrália/2020	Ensaio Teórico		Universidades e instituições de saúde.
E9	Eiff MP, Miller MF, Valenzuela S, Saseen JJ, Zierler B, Carraccio C, et al.^(29)^	EUA/2020	Métodos Mistos	Docentes da área de ciências da saúde e profissionais da saúde que atuam na educação.	Universidades e instituições de saúde.
E10	Fusco NM, Ohtake PJ.^(30)^	EUA/2019	Quase-Experimental	Docentes do curso de farmácia.	Universidades.
E11	Herinek D, Woodward-Kron R, Huber M, Helmer SM, Körner M, et al.^(31)^	Alemanha/2022	Quantitativo	Docentes e supervisores clínicos dos cenários de prática da área de ciências da saúde.	Universidades.
E12	Hudak NM, Ross E, Bynum W, Pasha N, Phillips B, Melcher BQ.^(32)^	EUA/2021	Métodos Mistos	Supervisores clínicos dos cenários de prática.	Instituições de saúde.
E13	Jacobs JL, Samarasekera DD, Chui WK, Chan SY, Wong LL, Liaw SY, et al.^(33)^	Ásia/2013	Qualitativo	Docentes da área de ciências da saúde.	Universidades.
E14	Julie H, Hess-April L, Wilkenson J, Cassiem W, Rhoda A.^(34)^	África do Sul/2016	Qualitativo	Docentes da área de ciências da saúde e supervisores clínicos dos cenários de prática.	Universidades e instituições de saúde.
E15	LeGros TA, Amerongen HM, Cooley JH, Schloss EP.^(35)^	EUA/2015	Métodos Mistos	Docentes da área de ciências da saúde e preceptores médicos.	Universidades e instituições de saúde.
E16	McCutcheon LRM, Whitcomb K, Craig DC, Klein MS, Burley H, Youngblood T, et al.^(36)^	EUA/2017	Quase-Experimental	Supervisores clínicos dos cenários de prática (profissionais dos cursos de farmácia).	Universidades e instituições de saúde.
E17	Mohammed CA, Chaturvedi A, Kamath MG, Ummer SV, Bajaj G.^(37)^	Índia/2023	Quantitativo	Docentes da área de ciências da saúde. Todos os participantes do programa de bolsas de aprimoramento docente em EIP.	Universidades e instituições de saúde.
E18	Poirier T, Wilhelm M.^(38)^	EUA/2014	Qualitativo	Docentes da área de ciências da saúde.	Universidades.
E19	Ratka A, Zorek JA, Meyer SM.^(39)^	EUA/2017	Revisão da Literatura Narrativa		
E20	Rider EA, Chou C, Abraham C, Weissmann P, Litzelman DR, Hatem D, et al.^(40)^	EUA/2023	Qualitativo	Docentes da área de ciências da saúde e supervisores clínicos dos cenários de prática.	Universidades e instituições de saúde.
E21	Schapmire TJ, Head BA, Furman CD, Jones C, Peters B, Shaw MA, et al.^(41)^	EUA/2021	Quantitativo	Docentes da área de ciências da saúde.	Universidades.
E22	Shelton LR, Thomason AR, Hensarling RW.^(42)^	EUA/2022	Quantitativo	Docentes da área de ciências da saúde, participantes do programa de bolsas de aprimoramento docente em EIP.	Universidades e instituições de saúde.
E23	Stokes CK.^(43)^	EUA/2021	Revisão de Literatura Narrativa		
E24	Thistlethwaite JE, Vlasses PH.^(44)^	Canadá/2021	Ensaio Teórico/ Capítulo de Livro		
E25	Waters L, Marrs SA, Tompkins CJ, Fix R, Finucane S, Coogle CL, et al.^(45)^	EUA/2022	Métodos Mistos	Docentes da área de ciências da saúde.	Universidades.
E26	Yorke AM, Hoelcher DC, Stalburg CM, Daniels T, Aebersold M, Patterson V, et al.^(46)^	EUA/2022	Métodos Mistos	Docentes da área de ciências da saúde.	Universidades.

Legenda: *ID = Identificação do estudo.

Fonte: Elaborado pelas autoras.

Na análise dos dados emergiram duas categorias sobre competências de desenvolvimento docente, que contribuíram para responder ao objetivo desta revisão de escopo: 1) Competências colaborativas interprofissionais e de facilitação para a EIP; e 2) Fundamentação pedagógica das ações de EIP: teorias, abordagens de aprendizagem, estratégias de ensino-aprendizagem e processos de avaliação desenvolvidos por docentes na EIP. Os temas que emergiram da análise de dados estão apresentados no Quadro 3 e sintetizados na Figura 2.

**Quadro 3:** Competências (conceito) e fundamentos teóricos para o desenvolvimento docente na educação interprofissional em saúde – São Carlos, SP, Brasil, 2024.

*ID	Competências (conceito)	Fundamentos teóricos		
		Teorias da aprendizagem	Ensino-Aprendizagem	Processo-Avaliativo
E1	Citou a comunicação, porém, não sinalizou quadro/estrutura de competências colaborativas.	Aprendizagem experiencial.	*Workshops*, métodos interativos, aprendizagem reflexiva, atividades em grupos interprofissionais, *Role-Play*, *feedback* e estratégias gerais de comunicação.	Instrumento qualitativo: análise temática indutiva com apoio da tipologia de Kirkpatrick. Instrumento quantitativo com escala *Likert*. Aplicação: antes e após a iniciativa para o desenvolvimento docente na EIP.
E2	Colaborativas, específicas e comuns^(47)^. Competências para a facilitação em EIP^(48)^. Competências de sensibilidade cultural e segurança^(49)^.	Aprendizagem experiencial.	Aprendizagem baseada em problemas.	Não aparece.
E3	Colaborativas^(15)^.	Não aparece.	Oficinas, *workshops* e aprendizagem em pequenos grupos.	Instrumento quantitativo pautado em escala validada^(50)^, escala de *Likert*. Aplicação: antes e após iniciativa para o desenvolvimento docente na EIP.
E4	Citou, porém, não sinalizou quadro/estrutura de competências colaborativas.	Aprendizagem baseada em competências.	Não aparece.	Não aparece.
E5	Colaborativas^(16)^.	Não aparece.	Atividades de quebra-gelo, reuniões, estudos de caso, simulações, módulos *on-line*, vídeos, *workshops* de mentoria, discussões em grupo, atividade de investigação apreciativa, reflexão e registros nos portfólios.	Instrumentos qualitativos: *feedback*, teste de conhecimento, lista de verificação observacionais, exercícios de reflexão, *debriefing* e entrevistas. Instrumentos quantitativos: questionários com escala *Likert.* Aplicação: os autores não reportaram.
E6	Colaborativas^(14)^.	Não aparece.	*Workshops* presenciais, atividades *on-line*, trabalho em pequenos grupos, exercícios de reflexão em pares, discussões em pequenos e grandes grupos.	Instrumentos quantitativos: Escala *Likert* e questionário validado^(51)^. Aplicação: antes e após a iniciativa para o desenvolvimento docente na EIP.
E7	Colaborativas^(15)^.	Não aparece.	Seminários e casos clínicos. Os autores relatam terem utilizado outras estratégias, porém, não houve detalhamento.	Instrumentos quantitativos como: escalas de autopercepção. Aplicação: antes e após a iniciativa para o desenvolvimento docente na EIP.
E8	Colaborativas^(14,16)^.	Aprendizagem significativa.	Aprendizagem em pequenos grupos, fóruns *on-line*, simulações interprofissionais, aprendizagem entre pares, atividades de reflexão, sala de aula invertida e momento de síntese ao final da aula.	Instrumento qualitativo: *feedback* entre pares. Aplicação: os autores não reportaram.
E9	Colaborativas^(15)^.	Não aparece.	Oficinas e *workshops.*	Instrumento qualitativo: observação participante. Instrumento quantitativo: questionários. Aplicação: antes, durante, no término e após um ano do encerramento da iniciativa para o desenvolvimento docente na EIP.
E10	Colaborativas^(16)^.	Aprendizagem experiencial.	Estudo de caso com a temática de comunicação.	Instrumento qualitativo: feedback. Instrumento quantitativo validado^(52)^. Aplicação: antes e após iniciativa para o desenvolvimento docente na EIP.
E11	Citou, porém, não sinalizou quadro/estrutura de competências colaborativas.	Não aparece.	Reuniões presencias, treinamento de habilidades (não houve detalhamento das habilidades) palestras *on-line*, *workshops* e discussão mediada pelos tutores.	Não aparece.
E12	Colaborativas^(16)^.	Aprendizagem experiencial e social.	Leitura prévias, apresentações seguidas de discussões para pequenos e grandes grupos, prática reflexiva, aprendizagem entre pares e prática de competências para facilitação.	Instrumento qualitativo: análise temática semântica. Instrumento quantitativo: questionários com escala *Likert*. Aplicação: antes e após a iniciativa para o desenvolvimento docente na EIP.
E13	Colaborativas^(16)^.	Não aparece.	Reuniões, *briefings*, palestras e *workshops*.	Instrumentos qualitativos: exercícios de reflexão e relatórios de grupo. Aplicação: não reportou os momentos de aplicação das avaliações.
E14	Colaborativas^(14)^.	Aprendizagem experiencial.	Oficinas, *workshop* e reuniões em pequenos grupos seguidas de apresentações.	Instrumentos qualitativos: discussões em pequenos grupos e análise temática. Aplicação: após a iniciativa para o desenvolvimento docente na EIP.
E15	Colaborativas^(15)^. Competências de Facilitação em EIP^(23,48)^. Competências de sensibilidade cultural e segurança^(49)^.	Aprendizagem de adultos, experiencial e construtivista.	Discussão em grupo, trocas de experiências e atividades de reflexão.	Instrumento qualitativo: *feedback* e *debriefing*. Instrumentos quantitativos: escala validada^(53)^. Aplicação: antes, durante, no término e após um mês do encerramento da iniciativa para o desenvolvimento docente na EIP.
E16	Colaborativas^(15)^.	Aprendizagem experiencial.	Modelo de Exercício de ensino estruturado por objetivos interprofissionais (iOSTE) e palestras.	Instrumento qualitativo: avaliação por rubrica e *debriefing.* Instrumento quantitativo: questionários. Aplicação: antes e após a iniciativa para o desenvolvimento docente na EIP.
E17	Citou, porém, não sinalizou quadro/estrutura de competências colaborativas.	Não aparece.	Aprendizagem baseada em projetos.	Instrumento quantitativo questionário validado^(54)^. Aplicação: antes e após a iniciativa para o desenvolvimento docente na EIP.
E18	Colaborativas^(15)^.	Aprendizagem experiencial.	Seminários, pôsteres, mesa redonda temática com aprendizagem ativa, simulações baseadas em casos e grupo de discussão colaborativo.	Instrumento qualitativo: questionários. Aplicação: antes, no dia da atividade e no término da iniciativa para o desenvolvimento docente na EIP.
E19	Colaborativas^(14)^.	Aprendizagem experiencial.	Palestras, *workshops*, seminários, estudos de caso, *Role-Play*, pacientes padronizados, discussões, pôsteres, plenário interativo e sessões de treinamentos *just-in-time.*	Instrumentos quantitativos: escalas validadas^(50,55)^. Aplicação: antes e após a iniciativa para o desenvolvimento docente na EIP.
E20	Colaborativas^(14,16)^.	Aprendizagem experiencial e significativa.	Atividades de aterramento *check-in e check-out*; pequenos grupos, círculo, frente a frente com interação entre si; escrita e leitura de investigação apreciativa reflexão narrativa com mediação de facilitador; reflexão baseada em casos-exercícios; *Role-Play* abordagem interprofissional para praticar habilidades de comunicação de más-notícias e divulgação de erros e plano de ação para desenvolver competências colaborativas^(47,48)^.	Instrumento qualitativo: questionário apreciativo com reflexão narrativa. Aplicação: após a iniciativa para o desenvolvimento docente na EIP.
E21	Colaborativas^(14,16)^.	Aprendizagem de adultos.	Diagnóstico situacional a fim de explorar o potencial de sucesso da difusão de inovações na instituição de origem do docente, *webinários,* módulos interativos *on-line*, treinamento para preencher instrumentos de planejamento, mentoria, *feedback,* aprendizagem baseada em problemas, relatórios de processos e vídeos.	Instrumento quantitativo: questionário validado^(56)^. Avaliação: antes e após a iniciativa para o desenvolvimento docente na EIP.
E22	Citou, porém, não sinalizou quadro/estrutura de competências colaborativas.	Não aparece.	*Workshop* e atividades *on-line* e simulação.	Instrumento quantitativo: Escala validada^(50)^. Avaliação: antes e após a iniciativa para o desenvolvimento docente na EIP.
E23	Citou, porém, não sinalizou quadro/estrutura de competências colaborativas.	Citou, porém, não detalhou.	A escrita intencional/pesquisa como estratégia de aprendizagem para desenvolver novos facilitadores, currículos/atividades de EIP, aplicação de princípios de aprendizagem ativa, orientação e liderança.	Instrumentos qualitativos: questionários para avaliar as mudanças de comportamentos baseados na tipologia Kirkpatrick. Aplicação: os autores não reportaram.
E24	Colaborativas^(14,16)^.	Aprendizagem de adultos.	Métodos interativos (não houve detalhamento).	Não aparece.
E25	Colaborativas^(16)^.	Aprendizagem experiencial.	Sessões em formato híbrido, atividades em pequenos grupos, estudo de caso e vídeos.	Instrumentos qualitativos: avaliação formativa, *debriefing* e grupos focais. Instrumentos quantitativos: avaliação somativa e questionários baseados nas competências colaborativas interprofissionais^(16)^. Avaliação: antes e após a iniciativa para o desenvolvimento docente na EIP.
E26	Colaborativas^(16)^ e uma competência adicional: Humildade intercultural.	Não aparece.	Atividades baseadas em estudo de casos.	Não aparece.

Legenda: *ID = Identificação do estudo.

Fonte: Elaborado pelas autoras.

**Figura 2:** Síntese dos resultados nas categorias de análise - São Carlos, SP, Brasil, 2024.

**Fonte:** Figura construída na ferramenta Canva pelas autoras.

Na primeira categoria, as competências mapeadas incluíram: competências colaborativas interprofissionais e de facilitação para a EIP. As competências colaborativas interprofissionais para a EIP foram abordadas de maneiras diversas nas publicações baseadas em referenciais teóricos consolidados^(14–16,47)^. O quadro canadense do CIHC^(14)^ foi mencionado em sete estudos dessa revisão^(26,28,34,39–41,44)^. O quadro estadunidense do IPEC^(15)^ versão publicada em 2011, foi utilizado em seis estudos incluídos^(24,27,29,35,36,38)^ e sua versão atualizada em 2016^(16)^ estava presente em 10 estudos^(25,28,30,32,33,40,44,45,46)^.

Apenas um estudo^(23)^ utilizou o modelo de três competências para orientar a EIP: 1) Competências comuns, conjunto de conhecimentos gerais para todas as profissões; 2) Competências complementares ou específicas, que diferenciam uma profissão da outra; e 3) Competências colaborativas, entre profissionais da mesma ou de diferente profissão, com inclusão de usuários, famílias e comunidades^(47)^. Alguns estudos aplicaram ambos os quadros^(14,16)^ para subsidiar seus estudos^(28,40,41,44)^. Ainda, seis estudos relataram a relevância das competências colaborativas interprofissionais mas não sinalizaram nenhum quadro ou estrutura para compor suas análises^(13,22,31,37,42,43)^, e um deles focou na comunicação^(22)^.

Dentre as competências interprofissionais colaborativas, 16 dos estudos incluídos destacaram os domínios do trabalho em equipe e da comunicação^(14,15,26,28,30,33–36,38–41,44–46)^ como parte relevante das intervenções realizadas, contudo não aprofundaram no detalhamento de atributos e desempenhos esperados.

Duas publicações^(23,35)^ incluíram referenciais teóricos sobre a facilitação na EIP^(48)^ e competências de sensibilidade cultural e segurança na EIP^(49)^, embora publicados anteriormente aos quadros/estruturas de competências colaborativas, apresentam características semelhantes.

As competências para a facilitação da EIP identificadas^(48)^ foram: 1) Compromisso com a EIP e PIC; 2) Credibilidade em relação à EIP; 3) Modelagem positiva de papéis; 4) Compreensão profunda dos métodos de aprendizagem interativa e confiança para sua aplicação; 5) Conhecimento de dinâmicas de grupo; 6) Confiança e flexibilidade para utilizar as diferenças profissionais em grupo e 7) Equilíbrio entre as necessidades individuais e as grupais, além de disposição ante às dificuldades.

As competências de sensibilidade cultural e segurança na EIP^(49)^ referem-se a: 1) Realizar esforços para modificar sua perspectiva de mundo; 2) Ter compreensão das questões culturais fundamentais; 3) Compreender a cultura dos profissionais com os quais trabalha; 4) Compreender as questões culturais fundamentais que dizem respeito ao processo saúde-doença; 5) Estabelecer relação de confiança com os usuários; 6) Proporcionar acolhimento no contexto da assistência de saúde; e 7) Negociar as intervenções de saúde, com o consentimento dos usuários.

A segunda categoria foi composta por publicações que apresentaram os fundamentos pedagógicos, teórico-metodológicos, teorias ou abordagens de aprendizagem, estratégias de ensino- aprendizagem e de avaliação, adotados pelos docentes. Das 26 publicações incluídas, 15 abordaram teorias de aprendizagem para orientar as iniciativas de desenvolvimento docente na EIP: aprendizagem experiencial^(22,23,30,34–36,38–40,45)^, aprendizagem de adultos^(35,41,44)^, aprendizagem significativa^(28,40)^, abordagem construtivista^(35)^, aprendizagem baseada em competências (ensino por competências)^(13)^ e aprendizagem social^(32)^.

A maioria dos estudos detalharam as estratégias de ensino e aprendizagem (Quadro 3), aspecto ausente em apenas uma publicação, na qual também não foram apresentados os instrumentos referentes ao processo avaliativo^(13)^. Ademais, 22 publicações descreveram diferentes metodologias de ensino-aprendizagem, ativas e interativas, que favorecem a troca de experiências entre docentes, supervisores, preceptores clínicos e profissionais da saúde^(22,24-36,38-45)^. Três pesquisas mencionaram apenas uma estratégia de ensino-aprendizagem para a oferta pedagógica^(13,37,46)^.

Os estudos utilizaram uma variedade de instrumentos qualitativos e quantitativos para avaliação, bem como diversificaram os períodos em que foram aplicados. Os instrumentos quantitativos de avaliação foram empregados em oito estudos, todos com dois momentos de aplicação, antes e após as iniciativas para o desenvolvimento da EIP^(24,26,27,30,37,39,41,42)^, entre as quais estão escalas do tipo *Likert* e questionários validados, como *The Interdisciplinary Education Perception Scale* ^(50)^, *The Interprofessional Education for Collaborative Patient-Centred Care Questionnaire* ^(51)^, *Interprofessional Collaborative Competencies Attainment Survey* ^(52)^, *Interprofessional Facilitation Scale* ^(53)^, *Perception of Interprofessional Collaboration Model Questionnaire* ^(54)^ *, Readiness for Interprofessional Learning Scale* ^(55)^ e *Core Competencies for Interprofessional Practice Individual Competency Assessment Tool* ^(56)^.

Os instrumentos qualitativos de avaliação foram questionários, entrevistas, exercícios de reflexão, relatórios de grupo, avaliação por rubrica, discussões em pequenos grupos interprofissionais, *feedback* presencial e autoavaliação. Seis estudos aplicaram instrumentos qualitativos para avaliar as iniciativas para o desenvolvimento docente na EIP isoladamente^(28,33,34,38,40,43)^. Dos estudos analisados, três não relataram o momento de aplicação dos instrumentos avaliativos^(28,33,43)^ e dois aplicaram somente após a iniciativa^(34,40)^. Um estudo que aplicou o instrumento de avaliação apenas ao final não detalhou os objetivos de aprendizagem, contudo, os participantes relataram reações positivas sobre a intervenção, mudança de atitudes e percepções, além de aquisição de conhecimentos e habilidades, de forma colaborativa^(34)^.

Ao todo, sete estudos aplicaram instrumentos para avaliação de abordagens qualitativa e quantitativa^(22,25,29,32,35,36,45)^. Destes, um não descreveu o momento de sua aplicação^(25)^, dois aplicaram em quatro momentos: antes, durante, ao final e algum tempo depois da intervenção de EIP^(29,35)^, sendo que um deles aplicou a avaliação um ano após a intervenção, embora os seus benefícios não tenham sido avaliados^(29)^. Os demais estudos aplicaram instrumentos em dois momentos distintos, antes e após a intervenção^(22,32,36,45)^.

## DISCUSSÃO

Esta revisão de escopo permitiu mapear as competências colaborativas interprofissionais que docentes, supervisores, preceptores clínicos, profissionais de saúde e educadores compreendem ser necessárias durante o processo de formação interprofissional. Também foram identificados os fundamentos pedagógicos, teórico-metodológicos, abarcados pelas teorias, abordagens de aprendizagem, estratégias de ensino-aprendizagem e de avaliação, que consistem em princípios viabilizadores de competências para o desenvolvimento docente na EIP. Optou-se por sistematizar os fundamentos pedagógicos referidos, considerando que o campo da EIP possui especificidade que requer a aplicação de estratégias ativas, interativas e compartilhadas de ensino-aprendizagem e de avaliação. Desse modo, os achados podem contribuir para instrumentalizar docentes no processo de planejamento, implementação e avaliação de propostas curriculares e iniciativas de EIP.

Destaca-se que majoritariamente as pesquisas identificadas utilizam os quadros CIHC e IPEC, demonstrando haver alinhamento das suas iniciativas aos quadros/estruturas da PIC^(14-16)^. Na presente revisão identificou-se fortemente os domínios da comunicação e do trabalho em equipe, que expressam a intencionalidade de fortalecer a força de trabalho em saúde na perspectiva interprofissional. Contudo, é relevante salientar que o compromisso da EIP também destaca o cuidado centrado nos usuários, famílias e comunidades, presente nesses quadros referenciais de análise e ainda incipientes nos estudos analisados.

Em relação à heterogeneidade de formação nas iniciativas de desenvolvimento docente em EIP, devem ser consideradas as particularidades e as características desses profissionais, como regime de trabalho parcial ou integral, qualificações e responsabilidades de ensino, condições que podem influenciar na abordagem e nos métodos de facilitação em EIP^(35)^. Além disso, docentes e demais facilitadores podem ter dificuldades para identificar oportunidades de aprendizagens em EIP nos cenários de prática, dado os diferentes repertórios e experiências prévias^(57)^. Nessa direção, estudo destacou que supervisores e preceptores clínicos identificaram oportunidades para a PIC, no entanto, docentes não tiveram a mesma percepção e necessitaram trabalhar em conjunto com esses agentes nos cenários de prática, para identificar e ampliar oportunidades formativas^(34)^.

A literatura sugere estratégias para lidar com essas dificuldades de percepção de docentes quanto às oportunidades reais da EIP. A designação de agentes experientes em facilitação em EIP pode proporcionar oportunidades de experimentação de diferentes métodos de ensino-aprendizagem e de dinâmicas de trabalho em equipe^(35)^. O envolvimento de agentes experientes em EIP, pode contribuir para a disseminação dos seus fundamentos pedagógicos e pressupostos, valorização dos diferentes saberes interprofissionais e fortalecimento de parcerias na integração ensino-serviço-comunidade, voltadas à construção de vivências e reflexões com ênfase na EIP, desde o início do percurso formativo.

Foi possível identificar e sintetizar teorias, abordagens de aprendizagem, estratégias de ensino-aprendizagem e de avaliação condizentes à EIP. Como destacado também nos resultados desta revisão, uma pesquisa revelou que muitas intervenções pedagógicas em EIP não apresentaram detalhamento preciso sobre a teoria de aprendizagem escolhida para guiar as intervenções^(58)^. Isso pode ocorrer devido à falta de entendimento acerca das teorias de aprendizagem empregadas nas iniciativas de EIP, mas também pode estar associada à falta de diretrizes pedagógicas, objetivos, resultados de aprendizagem e análise do impacto das iniciativas de EIP no corpo docente^(59,60)^. A ausência dessas teorias ou abordagens pedagógicas podem dificultar a construção e a implementação de iniciativas bem-sucedidas de desenvolvimento docente em EIP.

As teorias da aprendizagem selecionadas nos estudos foram aplicadas de maneira independente ou combinada. Três estudos utilizaram mais de uma teoria da aprendizagem para fundamentar as práticas pedagógicas, porém não descreveram sua articulação com os outros componentes pedagógicos e seus impactos^(32,35,40)^. As teorias da aprendizagem são aproximações parciais e restritas de aspectos e áreas específicas da aprendizagem, assim dificilmente constituem um conjunto completo de saberes que elucidem o significado dos fenômenos complexos que ocorrem durante a aprendizagem^(61)^. Se uma teoria ou abordagem não é suficiente para compreender os complexos fenômenos da aprendizagem, pode-se inferir que o planejamento pedagógico deve basear-se em mais de uma teoria ou abordagem, de modo que possa atender às diversas necessidades dos estudantes durante as atividades de EIP.

A teoria da aprendizagem de adultos destaca a responsabilidade e o protagonismo do sujeito acerca de seu próprio aprendizado, que no caso da EIP é também compartilhada, entre pessoas e grupos. Os princípios da educação de adultos enfatizam a aprendizagem cooperativa, colaborativa, reflexiva e socialmente construída entre pessoas^(62)^, condizente aos princípios e valores da EIP, que incluem colaboração, interação, respeito mútuo, parcerias e responsabilização^(63)^.

As experiências para o desenvolvimento docente na EIP também revelaram a abordagem construtivista, na qual o ser humano é visto como alguém que se transforma mediante relações e interações com o meio social e cultural no qual está inserido. Ou seja, aprendizagem e transformação mediante interações sociais^(64)^. A abordagem construtivista pode ser a base para elaboração, oferta e processo de avaliação de iniciativas de desenvolvimento docente na EIP, sendo que estudantes e profissionais de saúde precisam se relacionar constantemente. Assim, é necessário priorizar e incentivar experiências de aprendizagem que propiciem interações interprofissionais, entre profissionais, estudantes^(35,44)^ e desses com os usuários, famílias e comunidades.

Outra perspectiva apresentada nos resultados foi da aprendizagem experiencial, na qual o conhecimento é construído e modificado por meio das experiências. Dessa forma, o conhecimento não é considerado elemento imutável, como na abordagem tradicional. A experiência resulta da interação entre o mundo exterior e interno do ser, a partir de situações que permitem a criação e a recriação do conhecimento^(65)^. Durante as vivências, na construção e reconstrução de novos saberes, podem surgir conflitos que resultam em aprimoramento de percepções, compreensão e aquisição de novas competências. Nesse processo, os participantes da comunidade de aprendizagem trabalham em conjunto no entendimento dos fenômenos, na tomada de decisões e na resolução de problemas^(66)^, aspectos que reforçam a necessidade de vivências de EIP em cenários reais de práticas de saúde.

As publicações analisadas versaram também sobre abordagens ativas e interativas, dada a importância do intercâmbio intencional entre estudantes, educadores, supervisores, preceptores e docentes^(44,59)^, que se alinham aos princípios e valores da EIP. A metodologia ativa e interativa amplia a possibilidade de ensino e aprendizado crítico-reflexivo, comprometimento e protagonismo do estudante, oportunidades para aprimorar competências colaborativas^(63)^ e tornar o aprendizado mais atrativo e efetivo^(58)^.

Em suma, a maioria das pesquisas mencionaram as competências colaborativas interprofissionais oportunizadas por abordagem experiencial, entre outras teorias e fundamentações pedagógicas, de forma isolada ou complementar, como aprendizagem de adultos, significativa e socioconstrutivista. O elo comum entre essas abordagens é o deslocamento do protagonismo na aprendizagem, do docente para o estudante.

A literatura recomenda que as experiências de formação profissional em saúde apresentem as teorias que as fundamentam de forma mais explícita e robusta, objetivo da educação, a natureza do conhecimento, o que é valorizado e incluído no currículo, significado de aprender e como se avalia, papéis de professores e estudantes no processo, ou seja que haja alinhamento entre práticas de ensino, aprendizagem e avaliação aos valores e pressupostos paradigmáticos^(67)^. Há ainda pesquisa que indica o avanço da teorização da prática de ensino-aprendizagem imbuído de emoções, com reconhecimento de que a educação é para além do cognitivismo, um processo afetivo, social e político^(68)^. Outro estudo destacou a dificuldade de docentes apresentarem previamente a teoria que embasa as experiencias, indicada somente a posteriori^(69)^. Em revisão sobre intervenções de EIP, um conjunto de recomendações foi indicado para educadores projetarem, implementarem e avaliarem EIP, para alcance das competências interprofissionais. Especificamente em relação ao desenvolvimento docente, objeto de interesse desta revisão, identificaram que esses recebem formação antes do envolvimento na intervenção, mas não há informação se é específica para a facilitação da EIP ou de natureza pedagógica geral^(70)^.

Os estudos acima^(67–70)^ reforçam os achados desta revisão, fragilidades entre princípios teóricos e prática docente, experiências empíricas em detrimento de reflexão teórica mais profunda, falta de consenso sobre quais teorias estão na gênese da EIP e PIC e de como aplicá-las. As dificuldades elencadas expressam a complexidade na construção de experiencias formativas de EIP, razões que podem também se relacionar a tênue presença de teorias e fundamentos teóricos nas iniciativas de desenvolvimento docente em EIP.

Em relação ao processo avaliativo, o Centro para o Avanço da Educação Interprofissional (CAIPE, na sigla em inglês) propõe a implementação de estratégias dinâmicas, diversificadas e contínuas ao longo da ação em EIP^(63)^. O processo de avaliação deve ser pensado na elaboração do projeto, com definição dos instrumentos utilizados, alinhados aos objetivos, resultados da aprendizagem e momentos de sua aplicação^(71)^.

No que concerne à articulação da EIP para o fortalecimento das práticas dos sistemas de saúde, que no Brasil refere-se ao SUS, embora nenhum dos estudos tenha sido realizado no sistema de saúde brasileiro, as evidências inferem a responsabilidade das Instituições de Ensino Superior (IES), por meio de políticas e programas de desenvolvimento docente, cujo horizonte é a melhoria dos resultados em saúde, na perspectiva de interdependência entre sistema de saúde e de educação. O compromisso social das IES reside na formação da força de trabalho preparada para atender às necessidades dos usuários e dos sistemas de saúde, com ênfase na qualidade. A priorização de docentes e facilitadores no desenvolvimento de competências colaborativas interprofissionais viabiliza o fortalecimento do trabalho em equipe, da colaboração interprofissional e do cuidado centrado nos usuários.

Apesar do desafio da formação por competências colaborativas interprofissionais, integradas às necessidades das pessoas, famílias e comunidades e do reconhecimento da importância de avançar nessa direção, ainda persiste no cenário brasileiro o predomínio de cursos que não disponibilizam períodos no currículo para EIP e quando ocorrem, são atividades esporádicas e na perspectiva multiprofissional^(72)^.

A formação docente para a EIP tem o potencial de estimular o reconhecimento das oportunidades de formação interprofissional em contextos de prática, de oportunidades relevantes, que impactem no conhecimento, habilidades e na confiança no processo de ensino-aprendizagem^(73)^, cujo ponto de partida é a ocorrência de currículos mais flexíveis e articulados.

Embora o estudo tenha cumprido os procedimentos metodológicos preconizados para uma revisão de escopo, com amplo mapeamento da literatura e sem restrição temporal é importante considerar suas limitações. A restrição da busca em três idiomas, português, inglês e espanhol, pode ter provocado perdas, especialmente de publicações em outros idiomas. Somente uma publicação identificada como literatura cinza foi incluída nos achados, resultado que pode ser consequência das imprecisões em se mapear esse tipo de publicação. A heterogeneidade dos desenhos das publicações incluídas para a construção de sínteses e a falta da avaliação da qualidade metodológica dos estudos incluídos, etapa pouco presente em revisões de escopo.

## CONCLUSÃO

Esta revisão de escopo teve como objetivo mapear as competências de desenvolvimento docente para a EIP, mapeamento que demandou a análise das competências colaborativas interprofissionais e de facilitação presentes nas intervenções educativas, com destaque para os domínios de comunicação e trabalho em equipe.

As evidências produzidas destacam a necessidade de estudos futuros sobre o desenvolvimento de docentes e facilitadores de EIP com ênfase na integração ensino-serviço-comunidade, assim como na construção, disseminação e aplicação de um quadro de competências na perspectiva pedagógica em EIP. Também são necessários avanços em pesquisas que demonstrem as articulações entre as teorias/abordagens pedagógicas e as competências para o desenvolvimento docente na EIP, bem como os resultados das avaliações após as iniciativas, sobretudo a longo prazo.

Conclui-se na presente revisão de escopo que os domínios de competências para o desenvolvimento docente em EIP podem ser divididos em dois grupos: 1) Competências colaborativas interprofissionais e de facilitação para a EIP e 2) Fundamentos pedagógicos, teórico-metodológicos para o desenvolvimento docente na EIP: teorias, abordagens de aprendizagem, estratégias de ensino-aprendizagem e de avaliação. O presente mapeamento contribui para a fundamentação teórica no planejamento, implementação e avaliação de iniciativas para o desenvolvimento da EIP e de seus agentes, docentes, supervisores clínicos, preceptores dos cenários de prática, profissionais de saúde e dos próprios estudantes, para fortalecimento da interprofissionalidade.

Com base nos achados, recomenda-se que gestores de cursos de saúde, coordenadores de programas de desenvolvimento docente e formuladores de políticas educacionais reconheçam a importância das teorias de aprendizagem no fortalecimento da EIP e de desenvolvimento docente, com recomendação de que esses fundamentos/teorias pedagógicas sejam explicitados e estejam alinhados e coerentes às características da EIP. Ainda, recomenda-se investimento em espaços formativos, com ampla participação dos diferentes agentes corresponsáveis pela formação em saúde, para além do corpo docente.

## DISPONIBILIDADE DE DADOS

Todo o conjunto de dados que dá suporte aos resultados deste estudo está disponível mediante solicitação ao autor correspondente [Jaqueline Alcântara Marcelino da Silva].

## Footnotes

1 Nesta publicação são considerados docentes aqueles que planejam, implementam e avaliam as ações curriculares da formação em saúde e facilitadores, incluindo os demais agentes envolvidos na formação em saúde, tais como preceptores, supervisores, tutores, mentores que nem sempre participam de todas as etapas dos processos formativos.

# Translation: Competencias y fundamentos pedagógicos para el desarrollo docente en la educación interprofesional en salud: revisión de alcance*

**DOI:** 10.1590/1980-220X-REEUSP-2024-0421es

**Authors:** Daniella Rosaly Leite, Patrícia Silva Carneiro, Vivian Aline Mininel, Rosana Aparecida Salvador Rossit, Valéria Marli Leonello, Geisa Colebrusco de Souza Gonçalves, Jaqueline Alcântara Marcelino da Silva

**Affiliations:** 1 Universidade Federal de São Carlos, São Carlos, SP, Brasil.; 2 Universidade Federal de Goiás, Goiânia, GO, Brasil.; 3 Universidade Federal de São Paulo, São Paulo SP, Brasil.; 4 Universidade de São Paulo, São Paulo SP, Brasil.

**Keywords:** Competencia Profesional, Educación Basada en Competencias, Formación del Profesorado, Docentes, Educación Interprofesional

## Abstract

**Objetivo:**

Mapear las competencias para el desarrollo docente en la educación interprofesional en salud.

**Método:**

Se trata de una revisión de alcance, basada en las recomendaciones del Instituto Joanna Briggs con la pregunta orientadora: «¿Qué competencias se utilizan para el desarrollo docente en la educación interprofesional?». Las búsquedas se realizaron en las bases de datos MEDLINE/OVID, ERIC, CINAHL, Scopus, Web of Science, Google Scholar, EBSCO Open Dissertations, BDTD, Tesis y Disertaciones de CAPES, OpenGrey y MedNar, sin restricción temporal y en los idiomas español, portugués e inglés.

**Resultados:**

Se incluyeron 26 publicaciones, agrupadas en dos categorías: la primera se refiere a las competencias colaborativas interprofesionales y de facilitación para la educación interprofesional, con énfasis en los ámbitos de la comunicación y el trabajo en equipo. La segunda se refiere a la fundamentación pedagógica de la EIP, teorías, enfoques, estrategias de enseñanza-aprendizaje y evaluación, en las acciones de desarrollo docente para la educación interprofesional.

**Conclusión:**

Las evidencias mapeadas contribuyen a fortalecer las iniciativas de desarrollo docente y al avance de la fundamentación teórica y metodológica de la educación interprofesional en salud.

## INTRODUCCIÓN

Las organizaciones de salud han tenido que hacer frente a los retos que plantea la fragmentación de los sistemas de salud y de las prácticas asistenciales, que comprometen la disponibilidad, el acceso y la calidad de los servicios, así como a las dificultades inherentes a la formación de los profesionales de la salud. Existe una necesidad urgente de estrategias innovadoras, con énfasis en la elaboración de políticas y programas que consoliden la formación y el trabajo en el área de la salud^(1,2)^, ya que los servicios de salud requieren profesionales preparados a partir de competencias colaborativas interprofesionales para hacer frente a los cambios y desafíos de la atención sanitaria^(2)^.

La educación interprofesional en salud (EIP) busca contribuir a la reformulación de las matrices curriculares de los cursos de licenciatura y posgrado, con el objetivo de preparar a los profesionales para trabajar en equipo de manera eficiente, con enfoque en la interacción, la integración y la colaboración^(3)^, cuya atención se centre en la persona, el grupo o la población^(4)^. La EIP es un movimiento global y, en el contexto brasileño, encuentra posibilidades de aplicación en los servicios del Sistema Único de Salud (SUS), en diferentes espacios formativos: licenciatura, residencias y educación permanente en salud. En dichos contextos formativos, la EIP contribuye a movilizar reflexiones sobre el proceso de trabajo y la coproducción orientada al fortalecimiento de las competencias colaborativas interprofesionales y de prácticas comprometidas con las necesidades de salud de los usuarios y de la población, como protagonistas de la atención^(5)^.

Con la expansión de la EIP, se vuelve crucial preparar a los docentes para lidiar con los desafíos de este enfoque educativo innovador^(2,6)^. Se requiere que los docentes lideren, faciliten y elaboren planes de estudio de EIP, pero tienen dificultades para asumir estas funciones y responsabilidades^(7)^, ya que no han estado expuestos a actividades de aprendizaje y prácticas interprofesionales en su proceso de formación profesional o docente^(8)^, con escasas iniciativas de desarrollo docente, sobre todo desde la perspectiva de la EIP.

Aunque el término docente se utiliza ampliamente para referirse a los profesores universitarios, estos no son los únicos agentes que participan en la formación en salud. Se está ampliando la comprensión de que los profesionales que trabajan en los servicios de la red de atención también deben participar en la formación para el desarrollo de acciones en EIP, con reconocimiento de su contribución a la formación profesional^(9)^. De este modo, se reconoce que en las iniciativas de EIP participan facilitadores que son profesionales de la salud, doctores, maestros, especialistas en formación, preceptores, tutores y supervisores, que actúan como agentes corresponsables de la formación en salud y necesitan oportunidades formativas para actuar en la enseñanza en salud.

Un estudio de revisión sistemática indicó la necesidad de adoptar iniciativas para el desarrollo docente en la EIP, ya que entre los principales aspectos para una intervención interprofesional exitosa se encuentran la capacidad de facilitación de los docentes, la comprensión del enfoque educativo, las estrategias de enseñanza-aprendizaje, los procesos evaluativos y las competencias en la EIP^(10)^. En la misma línea, un estudio señaló que la práctica de la EIP enfatizó las competencias colaborativas interprofesionales para los estudiantes, pero se descuidaron las competencias docentes de facilitación^(11)^.

Así, el éxito de una intervención educativa requiere objetivos de aprendizaje, resultados esperados, contenidos, estrategias de enseñanza-aprendizaje y evaluación, además de las competencias a desarrollar, encadenadas de forma coherente^(12)^. Para que la planificación, implementación y evaluación de los currículos se basen en la EIP, los docentes necesitan oportunidades para desarrollar nuevas competencias^(7,10,13)^.

Ante lo anterior y ante la ausencia de estudios previos, se identificó la necesidad de producir un mapeo de las competencias para el desarrollo docente en la EIP, con la intención de minimizar las lagunas encontradas en la literatura, orientar políticas y programas en el contexto local (institucional) y en el macrocontexto de la formación en salud (sistema), para subsidiar futuras iniciativas en la temática. El objetivo de esta revisión de alcance fue mapear las competencias para el desarrollo docente en la EIP.

## REFERENCIA TEÓRICA

En la literatura, destacan dos cuadros que describen las competencias colaborativas interprofesionales, uno canadiense^(14)^y otro estadounidense^(15)^, que, aunque no abordan el desarrollo docente, indican las competencias para los profesionales que trabajan en los sistemas de salud. El cuadro de la *Canadian Interprofessional Health Collaborative* (CIHC)^(14)^ abarca cuatro competencias: claridad de roles; funcionamiento en equipo; resolución de conflictos interprofesionales; y liderazgo colaborativo, dependientes de dos ámbitos de competencias principales: comunicación interprofesional y atención centrada en los usuarios, las familias y las comunidades.

El marco canadiense tiene como objetivo identificar los conocimientos, habilidades, actitudes, comportamientos y juicios necesarios para alcanzar la excelencia y aplicarlos en situaciones de colaboración interprofesional. De este modo, estas competencias pueden contribuir a la planificación de las acciones de EIP, elaboradas por docentes y otros facilitadores^1^, con el fin de lograr acciones educativas exitosas^(14)^.

En 2024, el marco CIHC se actualizó con cambios en la terminología destinados a mejorar la colaboración interprofesional. Entre los cambios, cabe destacar la inclusión de los socios de los cuidados y los servicios de salud, así como de todas las personas involucradas en las relaciones de cuidado. El objetivo de la actualización fue mejorar la atención sanitaria ofrecida, mediante asociaciones colaborativas y centradas en las relaciones, además de facilitar la toma de decisiones conjuntas sobre las necesidades sociales y sanitarias. En la base del cuadro se presentaron los factores que influyen en la aplicación de las competencias, tales como la inclusión, el acceso y la equidad, relacionados con la complejidad y el contexto de la atención y los servicios sanitarios^(4)^.

El marco estadounidense, *Interprofessional Education Collaborative* (IPEC)^(15)^, presenta competencias esenciales para la práctica colaborativa. Publicado en 2011, con cuatro áreas temáticas relacionadas con la EIP: valores y ética; funciones y responsabilidades; comunicación interprofesional; equipo y trabajo en equipo^(15)^, fue actualizado en 2016, destacando el cambio de presentar las cuatro áreas temáticas mencionadas en un único dominio: competencia colaborativa interprofesional, con énfasis en la centralidad de la atención en los usuarios, las familias y las comunidades^(16)^.

La última versión del marco IPEC publicada en 2023^(17)^ expresa la práctica interprofesional colaborativa (PIC) como esencial para lograr mejores resultados en salud, desde la perspectiva de una atención segura, de calidad, accesible, equitativa y centrada en la persona. Se mantuvo el dominio singular de la competencia colaborativa interprofesional y las cuatro áreas (valores y ética; roles y responsabilidades; comunicación; equipos y trabajo en equipo) con la indicación del compromiso de la formación en salud a lo largo de la vida, con el fin de que los profesionales de la salud recién formados y con experiencia se comprometan con la construcción de dichas competencias.

## MÉTODO

### Tipo de Estudio

Se trata de una revisión de alcance (*scoping review*) basada en las recomendaciones del Instituto Joanna Briggs (JBI) y en los *Elementos preferidos para la presentación de informes sistemáticos y metaanálisis. Extensión para revisiones de alcance* (PRISMA) - *Extensión para revisiones de alcance* (PRISMA-ScR): *Lista de verificación y explicación* ^(18)^. La revisión de alcance tiene como objetivo mapear los principales conceptos que sustentan un área determinada del conocimiento, examinar la extensión y el alcance del objeto de investigación, fundamentar y divulgar los datos, así como identificar las lagunas existentes^(19)^. El protocolo fue elaborado y revisado por los autores y registrado prospectivamente en la plataforma *Open Science Framework* (OSF): https://osf.io/6gazx/. El estudio se llevó a cabo en etapas secuenciales: identificación de la pregunta de investigación, extracción, análisis y presentación de los resultados^(19)^. Durante el proceso, los autores mantuvieron debates para alcanzar alineamientos y consensos. 

### Pregunta de Investigación

Para la construcción de la pregunta de investigación se utilizó la estrategia mnemónica Población, Concepto y Contexto (PCC). Esta revisión tuvo como población (P) docentes, preceptores, tutores, mentores, profesores o facilitadores de la educación superior de los cursos de ciencias de la salud, el concepto (C), competencias relacionadas con el desarrollo docente para la EIP y el contexto (C) universidades y/o escenarios de prácticas de la formación en salud. De este modo, se adoptó la siguiente pregunta orientadora: «¿Qué competencias se utilizan para el desarrollo docente en la EIP?».

### Criterios de Elegibilidad

Se incluyeron estudios teóricos (ensayos teóricos y revisiones de la literatura), estudios empíricos primarios y secundarios, cuantitativos, cualitativos mixtos y cuasi experimentales publicados en los idiomas portugués, español e inglés, sin restricción de tiempo, con una población compuesta por docentes de educación superior en el área de las ciencias de la salud y preceptores de campo que abordaran las competencias necesarias para el desarrollo docente en el contexto de la EIP. Por lo tanto, en esta revisión consideramos, además de los docentes, a todos los profesionales de la salud que participan en la formación de los estudiantes (doctores, maestros, especialistas, licenciados), preceptores, supervisores y facilitadores que participan en la formación en salud. La literatura gris, como disertaciones, tesis, libros y/o capítulos de libros y manuales, se incluyó cuando respondía a la pregunta de la investigación. Los criterios de exclusión incluyeron reseñas, editoriales, actas de congresos y eventos.

### Fuentes de Datos y Estrategia de Búsqueda

La estrategia de búsqueda fue elaborada por los autores del estudio con el apoyo de una bibliotecaria experta, a partir de una combinación de Descriptores en Ciencias de la Salud (DeCS), Medical Subject Headings (MeSH) y operadores booleanos AND y OR, presentados en el Cuadro 1.

**Cuadro 1:** Estrategia de búsqueda en las bases de datos – São Carlos, SP, Brasil, 2024.

Base	Expresión
MEDLINE/OVID	**S1** (Faculty* OR Mentor* OR Professor* OR Tutor* OR Teacher* OR Preceptor* OR Instructor* OR Facilitat* OR Development).mp. [mp = title, abstract, original title, name of substance word, subject heading word, floating sub-heading word, keyword heading word, organism supplementary concept word, protocol supplementary concept word, rare disease supplementary concept word, unique identifier, synonyms] **S2** (Competency Based Education OR Competency Based Education OR Education, Competency-Based OR Competency-Based Educations OR Education, Competency Based OR Educations, Competency-Based OR Competency).mp. [mp = title, abstract, original title, name of substance word, subject heading word, floating sub-heading word, keyword heading word, organism supplementary concept word, protocol supplementary concept word, rare disease supplementary concept word, unique identifier, synonyms] **S3** (Interprofessional education OR interprofessional education OR IPE OR multi-professional education OR multi-professional education OR multi-professional education OR multidisciplinary education OR multi-disciplinary education).mp. [mp = title, abstract, original title, name of substance word, subject heading word, floating sub-heading word, keyword heading word, organism supplementary concept word, protocol supplementary concept word, rare disease supplementary concept word, unique identifier, synonyms] **S4** (1 and 2 and 3) ((Faculty* OR Mentor* OR Professor* OR Tutor* OR Teacher* OR Preceptor* OR Instructor* OR Facilitat* OR Development) AND (Competency Based Education OR Competency Based Education OR Education, Competency-Based OR Competency-Based Educations OR Education, Competency Based or Educations, Competency-Based OR Competency) AND (Interprofessional education OR interprofessional education OR IPE OR multi-professional education OR multi-professional education OR multi-professional education or multidisciplinary education OR multi-disciplinary education)).mp. [mp=title, book title, abstract, original title, name of substance word, subject heading word, floating sub-heading word, keyword heading word, organism supplementary concept word, protocol supplementary concept word, rare disease supplementary concept word, unique identifier, synonyms, population supplementary concept word, anatomy supplementary concept word]
ERIC y CINAHL	**S1** (Faculty* OR Mentor* OR Professor* OR Tutor* OR Teacher* OR Preceptor* OR Instructor* OR Facilitat* OR Development) AND (Competency Based Education OR Competency Based Education OR Education, Competency-Based OR Competency-Based Educations OR Education, Competency Based OR Educations, Competency-Based OR Competency) AND (Interprofessional education OR interprofessional education OR IPE OR multi-professional education OR multi-professional education OR multi-professional education OR multidisciplinary education OR multi-disciplinary education)
Web of Science	**S1** (Faculty* OR Mentor* OR Professor* OR Tutor* OR Teacher* OR Preceptor* OR Instructor* OR Facilitat* OR Development) AND (“Competency-Based Education” OR “Competency Based Education” OR “Education, Competency-Based” OR “Competency-Based Educations” OR “Education, Competency Based” OR “Educations, Competency-Based” OR Competency) AND (“Interprofessional education” OR “interprofessional education” OR IPE OR “multiprofessional education” OR “multi-professional education” OR “multiprofessional education” OR “multidisciplinary education” OR “multi-disciplinary education”)
Scopus	**S1** TITLE-ABS-KEY Faculty* OR Mentor* OR Professor* OR Tutor* OR Teacher* OR Preceptor* OR Instructor* OR Facilitat* OR Development) AND (Competency Based Education OR Competency Based Education OR Education, Competency-Based OR Competency-Based Educations OR Education, Competency Based OR Educations, Competency-Based OR Competency) AND (Interprofessional education OR interprofessional education OR IPE OR multi-professional education OR multi-professional education OR multi-professional education OR multidisciplinary education OR multi-disciplinary education)
Google Scholar	**S1** (Faculty* OR Mentor* OR Professor* OR Tutor* OR Teacher* OR Preceptor* OR Instructor* OR Facilitat* OR Development) AND (Competency Based Education OR Competency Based Education OR Education, Competency-Based OR Competency-Based Educations OR Education, Competency Based OR Educations, Competency-Based OR Competency) AND (Interprofessional education OR interprofessional education OR IPE OR multi-professional education OR multi-professional education OR multi-professional education OR multidisciplinary education OR multi-disciplinary education)
EBSCO *Open Dissertations*	**S1** (Faculty* OR Mentor* OR Professor* OR Tutor* OR Teacher* OR Preceptor* OR Instructor* OR Facilitat* OR Development) AND (Competency Based-Education OR Competency Based Education OR Education, Competency-Based OR Competency-Based Educations OR Education, Competency Based OR Educations, Competency-Based OR Competency) AND (Interprofessional education OR interprofessional education OR IPE OR multi-professional education OR multi-professional education OR multi-professional education OR multidisciplinary education OR multi-disciplinary education)
BDTD/ Teses e dissertações da CAPES	**S1** “Professor AND Competências AND Educação Interprofissional”
MedNar	**S1** “Faculty AND Competency AND Interprofessional Education Development”
OpenGrey	**S1** “Faculty AND Interprofessional”

Fuente: Elaborado por los autores.

Las búsquedas se realizaron en diciembre de 2023 en las siguientes bases de datos: *Medical Literature Analysis and Retrieval System* (MEDLINE/OVID), *Education Resources Information Center* (ERIC), *Cumulative Index to Nursing and Allied Health Literature* (CINAHL) a través de EBSCO, *Web of Science Institute for Scientific Information,* Scopus *SciVerse*, *Google Scholar*, EBSCO *Open Dissertations*, Catálogo de Tesis y Disertaciones (CTD) de la Coordinación de Perfeccionamiento del Personal de Nivel Superior (CAPES), Biblioteca Digital Brasileña de Tesis y Disertaciones (BDTD), *Deep Web Search Engine* (MedNar) y *System for Information on Grey Literature in Europe* (OpenGrey). Para identificar otros estudios elegibles, se realizaron búsquedas manuales en las referencias de los estudios seleccionados.

### Seleção dos Estudos

Os resultados obtidos foram exportados para o *software Rayyan*®^(20)^, para proceder a remoção automática dos estudos duplicados. A triagem e a seleção dos estudos foram realizadas mediante procedimento de duplo cego, por dois revisores independentes, em duas etapas, a partir da seleção por títulos e resumos, na primeira etapa, e por leitura dos textos completos, na segunda. As divergências na seleção foram resolvidas por terceiro revisor. As listas de referências dos estudos selecionados na segunda etapa também passaram pela análise do terceiro revisor para confirmar a pertinência de inclusão dos estudos sobre a temática. Os resultados das buscas e os estudos selecionados estão detalhados no fluxograma *Preferred Reporting Items for Systematic Reviews and Meta-Analyses Extension for Scoping Reviews* (PRISMA-ScR)^(18)^, apresentado a seguir:

### Análisis de los Datos y Síntesis de los Resultados

Para extraer los datos de los estudios seleccionados, se elaboró una hoja de cálculo en el *software Microsoft Excel®*. Se seleccionó la siguiente información relevante de los estudios: 1) Caracterización: autoría, país, año, enfoque de la publicación, población y contexto; 2) Identificación del estudio, competencias colaborativas interprofesionales, teorías o enfoques de enseñanza-aprendizaje y proceso evaluativo. 

Los datos se analizaron mediante un análisis de contenido temático descriptivo y deductivo^(19)^, que consistió en la organización de la información en tres etapas: preanálisis; exploración del material; e interpretación de los resultados^(21)^, correspondientes a las tres etapas del análisis de contenido en revisiones de alcance: preparación, organización y elaboración de informes^(19)^. Así, el material seleccionado se organizó y codificó en unidades de registro (temas) e indicadores no frecuenciales, seguido de la categorización y clasificación de los temas por diferenciación y reagrupamiento^(21)^. El análisis deductivo se basó en los constructos teóricos de la EIP y en los cuadros de competencias del CIHC^(14)^ y el IPEC^(15-17)^.

## RESULTADOS

Las búsquedas totalizaron 1776 registros, 26 estudios cumplieron los criterios de elegibilidad y se incluyeron en esta revisión, como se muestra en la Figura 1. Se publicaron entre los años 2009 y 2023, procedentes de los siguientes países: Estados Unidos (n = 15; 57 %), Canadá (n = 3; 11 %), Sudáfrica (n = 2; 8 %), Australia (n = 2; 8 %), Alemania (n = 1; 4 %), Arabia Saudita (n = 1; 4 %), Asia (n = 1; 4 %) e India (n = 1; 4 %). La mayoría eran investigaciones de método mixto (n = 8; 31%), cualitativas (n = 5; 19%), cuantitativas (n = 4; 15%), cuasi-experimentales (n = 4; 15%), revisión de literatura narrativa (n = 3; 12%) y ensayo teórico (n = 2; 8%). 

**Figura 1:** Flujograma PRISMA- ScR, identificación, selección y selección de los estudios incluidos en la revisión del alcance – São Carlos, SP, Brasil, 2024.

**Fuente:** Elaborado por los autores.

En cuanto al contexto de los estudios, 11 (42 %) se llevaron a cabo en instituciones de educación superior (IES), 10 (39 %) en instituciones de salud e IES, uno (4 %) en la Red de Educación Interprofesional de Sudáfrica y cuatro (15 %) no tenían un contexto definido, ya que eran ensayos teóricos o revisiones narrativas.

Como participantes, los estudios incluyeron docentes, supervisores clínicos y preceptores de los escenarios de práctica, profesionales de la salud involucrados en la formación y especialistas en interprofesionalidad, que trabajan en el área de las ciencias de la salud. Los datos extraídos de las publicaciones se presentan en la Cuadro 2.

**Cuadro 2:** Caracterización de los estudios egún autor, país, año de publicación, enfoque de la publicación, población y contexto – São Carlos, SP, Brasil, 2024.

*ID	Autoría	País/Año	Abordaje de la publicación	Población	Contexto
E1	Attrill S, Dymmott A, Wray A.^(22)^	Australia/2019	Cuantitativo y Cualitativo	Supervisores clínicos dos escenarios de práctica.	Universidades e instituciones de salud.
E2	Banfield V, Lackie K.^(23)^	Canadá/2009	Revisión de la Literatura Narrativa		
E3	Bashatah AS, Al-Ahmary KA, Arifi MA, Asiri YA, Alruthia Y, Metwally AS, et al.^(24)^	Arabia Saudita/2020	Cuasi-Experimental	Docentes del área de ciencias de la salud	Universidades.
E4	Botma Y.^(13)^	África do Sul/2019	Cualitativo	Expertos miembros de la Red de Educación Interprofesional de África Subsahariana.	*Workgroup of the Sub-Saharan Africa Interprofessional Education Network* (AfrIPEN).
E5	Brashers V, Haizlip J, Owen JA.^(25)^	EUA/2019	Métodos Mixtos	Docentes y supervisores clínicos de los escenarios de práctica del área de ciencias de la salud.	Universidades.
E6	Christianson TM, Bainbridge L, Halupa C.^(26)^	Canadá/2018	Métodos Mixtos	Docentes del área de ciencias de la salud.	Universidades.
E7	Coogle LC, Hackett L, Owens MG, Ansello EF, Mathews JH.^(27)^	EUA/2016	Cuasi-Experimental	Docentes del área de ciencias de la salud.	Universidades.
E8	Diggele CV, Roberts C, Burgess A.^(28)^	Australia/2020	Ensayo Teórico		Universidades e instituciones de salud.
E9	Eiff MP, Miller MF, Valenzuela S, Saseen JJ, Zierler B, Carraccio C, et al.^(29)^	EUA/2020	Métodos Mixtos	Docentes del área de ciencias de la salud y profesionales de la salud que trabajan en el ámbito de la educación.	Universidades e instituciones de salud.
E10	Fusco NM, Ohtake PJ.^(30)^	EUA/2019	Cuasi-Experimental	Docentes do curso de farmacia.	Universidades.
E11	Herinek D, Woodward-Kron R, Huber M, Helmer SM, Körner M, et al.^(31)^	Alemania/2022	Cuantitativo	Docentes e supervisores clínicos de los escenarios de práctica del área de ciencias de la salud.	Universidades.
E12	Hudak NM, Ross E, Bynum W, Pasha N, Phillips B, Melcher BQ.^(32)^	EUA/2021	Métodos Mixtos	Supervisores clínicos de los escenarios de práctica.	Instituciones de salud.
E13	Jacobs JL, Samarasekera DD, Chui WK, Chan SY, Wong LL, Liaw SY, et al.^(33)^	Asia/2013	Cualitativo	Docentes del área de ciencias de la salud.	Universidades.
E14	Julie H, Hess-April L, Wilkenson J, Cassiem W, Rhoda A.^(34)^	África do Sul/2016	Cualitativo	Docentes del área de ciencias de la salud y supervisores clínicos de los escenarios de práctica.	Universidades e instituciones de salud.
E15	LeGros TA, Amerongen HM, Cooley JH, Schloss EP.^(35)^	EUA/2015	Métodos Mixtos	Docentes del área de ciencias de la salud e preceptores médicos.	Universidades e instituciones de salud.
E16	McCutcheon LRM, Whitcomb K, Craig DC, Klein MS, Burley H, Youngblood T, et al.^(36)^	EUA/2017	Cuasi-Experimental	Supervisores clínicos de los escenarios de práctica (profesionales dos cursos de farmacia).	Universidades e instituciones de salud.
E17	Mohammed CA, Chaturvedi A, Kamath MG, Ummer SV, Bajaj G.^(37)^	India/2023	Cuantitativo	Docentes del área de ciencias de la salud. Todos los participantes del programa de becas de mejoramiento docente en EIP.	Universidades e instituciones de salud.
E18	Poirier T, Wilhelm M.^(38)^	EUA/2014	Cualitativo	Docentes del área de ciencias de la salud.	Universidades.
E19	Ratka A, Zorek JA, Meyer SM.^(39)^	EUA/2017	Revisión de la Literatura Narrativa		
E20	Rider EA, Chou C, Abraham C, Weissmann P, Litzelman DR, Hatem D, et al.^(40)^	EUA/2023	Cualitativo	Docentes del área de ciencias de la salud y supervisores clínicos de los escenarios de práctica.	Universidades e instituciones de salud.
E21	Schapmire TJ, Head BA, Furman CD, Jones C, Peters B, Shaw MA, et al.^(41)^	EUA/2021	Cuantitativo	Docentes del área de ciencias de la salud	Universidades.
E22	Shelton LR, Thomason AR, Hensarling RW.^(42)^	EUA/2022	Cuantitativo	Docentes del área de ciencias de la salud, programa de becas de mejoramiento docente en EIP.	Universidades e instituciones de salud.
E23	Stokes CK.^(43)^	EUA/2021	Revisión de la Literatura Narrativa		
E24	Thistlethwaite JE, Vlasses PH.^(44)^	Canadá/2021	Ensayo Teórico/ Capítulo de Libro		
E25	Waters L, Marrs SA, Tompkins CJ, Fix R, Finucane S, Coogle CL, et al.^(45)^	EUA/2022	Métodos Mixtos	Docentes del área de ciencias de la salud	Universidades.
E26	Yorke AM, Hoelcher DC, Stalburg CM, Daniels T, Aebersold M, Patterson V, et al.^(46)^	EUA/2022	Métodos Mixtos	Docentes del área de ciencias de la salud	Universidades.

Siglas: *ID = Identificación del estudio.

Fuente: Elaborado por las autoras.

En el análisis de los datos surgieron dos categorías sobre competencias de desarrollo docente, que contribuyeron a responder al objetivo de esta revisión de alcance: 1) Competencias colaborativas interprofesionales y de facilitación para la EIP; y 2) Fundamentación pedagógica de las acciones de EIP: teorías, enfoques de aprendizaje, estrategias de enseñanza-aprendizaje y procesos de evaluación desarrollados por docentes en la EIP. Los temas que surgieron del análisis de los datos se presentan en el Cuadro 3 y se sintetizan en la Figura 2.

**Cuadro 3:** Competencias (concepto) y fundamentos pedagógicos para el desarrollo docente en la educación interprofesional en salud – São Carlos, SP, Brasil, 2024.

*ID	Competencias (concepto)	Fundamentos teóricos		
		Teorías del aprendizaje	Enseñanza-Aprendizaje	Proceso-Evaluativo
E1	Mencionó la comunicación, pero no señaló un marco o estructura de competencias colaborativas.	Aprendizaje experiencial.	Workshops, métodos interactivos, Aprendizaje reflexiva, actividades em grupos interprofesionales, Role-Play, feedback y estrategias genéricas de comunicación.	Instrumento cualitativo: análisis temático inductivo con el apoyo de la tipología de Kirkpatrick. Instrumento cuantitativo con escala de Likert. Aplicación: antes y después de la iniciativa para el desarrollo docente en la EIP.
E2	Colaborativas, específicas y comunes^(47)^. Competencias para la facilitación en EIP^(48)^. Competencias de sensibilidad cultural y seguridad^(49)^.	Aprendizaje experiencial.	Aprendizaje basado en problemas.	No aparece.
E3	Colaborativas^(15)^.	No aparece.	Oficinas, workshops e Aprendizaje en pequeños grupos.	Instrumento Cuantitativo pautado em escala validada^(50)^, escala de Likert. Aplicación: antes y después de la iniciativa para el desarrollo docente en la EIP.
E4	Mencionó pero no señaló un marco o estructura de competencias colaborativas.	Aprendizaje basado en competencias.	No aparece.	No aparece.
E5	Colaborativas^(16)^.	No aparece.	Actividades para romper el hielo, reuniones, estudios de casos, simulaciones, módulos en línea, vídeos, talleres de mentoría, debates en grupo, actividades de investigación apreciativa, reflexión y registros en los portafolios.	Instrumentos cualitativos: feedback, prueba de conocimientos, lista de verificación observacional, ejercicios de reflexión, debriefing y entrevistas. Instrumentos cuantitativos: cuestionarios con escala Likert. Aplicación: los autores no lo han informado.
E6	Colaborativas^(14)^.	No aparece.	Talleres presenciales, actividades en línea, trabajo en pequeños grupos, ejercicios de reflexión en parejas, debates en grupos pequeños y grandes.	Instrumentos cuantitativos: Escala de Likert y cuestionario validado^(51)^. Aplicación: antes y después de la iniciativa para el desarrollo docente en la EIP.
E7	Colaborativas^(15)^.	No aparece.	Seminarios y casos clínicos. Los autores informan haber utilizado otras estrategias, pero no proporcionan detalles.	Instrumentos cuantitativos como: escalas de autopercepción. Aplicación: antes y después de la iniciativa para el desarrollo docente en la EIP.
E8	Colaborativas^(14,16)^.	Aprendizaje significativa.	Aprendizaje en grupos pequeños, foros en línea, simulaciones interprofesionales, aprendizaje entre pares, actividades de reflexión, aula invertida y momento de síntesis al final de la clase.	Instrumento Cualitativo: feedback entre pares. Aplicación: los autores no informaron al respecto.
E9	Colaborativas^(15)^.	No aparece.	Oficinas e workshops.	Instrumento cualitativo: observación participante. Instrumento cuantitativo: cuestionarios. Aplicación: antes, durante, al final y un año después de la finalización de la iniciativa para el desarrollo docente en la EIP.
E10	Colaborativas^(16)^.	Aprendizaje experiencial.	Estudio de caso con la temática de la comunicación.	Instrumento Cualitativo: feedback. Instrumento Cuantitativo validado^(52)^. Aplicación: antes y después de iniciativa para desarrollo docente en EIP.
E11	Mencionó pero no señaló un marco o estructura de competencias colaborativas.	No aparece.	Reuniones presenciales, capacitación en habilidades (no se detallaron las habilidades), conferencias en línea, talleres y debates moderados por los tutores.	No aparece.
E12	Colaborativas^(16)^.	Aprendizaje experiencial y social.	Lecturas previas, presentaciones seguidas de debates para grupos pequeños y grandes, práctica reflexiva, aprendizaje entre pares y práctica de competencias para la facilitación.	Instrumento cualitativo: análisis temático semántico. Instrumento cuantitativo: cuestionarios con escala de Likert. Aplicación: antes y después de la iniciativa para el desarrollo docente en la EIP.
E13	Colaborativas^(16)^.	No aparece.	Reuniones, sesiones informativas, conferencias y talleres de trabajo.	Instrumentos cualitativos: ejercicios de reflexión e informes de grupo. Aplicación: No informó sobre los momentos de aplicación de las evaluaciones.
E14	Colaborativas^(14)^.	Aprendizaje experiencial.	Talleres, workshops y reuniones en grupos reducidos seguidos de presentaciones.	Instrumentos cualitativos: debates en pequeños grupos y análisis temático. Aplicación: tras la iniciativa para el desarrollo docente en la EIP.
E15	Colaborativas^(15)^. Competencias de facilitación en EIP^(23,48)^. Competencias de sensibilidad cultural y seguridad^(49)^.	Aprendizaje de adultos, experiencial y constructivista.	Debate en grupo, intercambio de experiencias y actividades de reflexión.	Instrumento cualitativo: feedback y debriefing. Instrumentos cuantitativos: escala validada^(53)^. Aplicación: antes, durante, al término y un mes después de la finalización de la iniciativa para el desarrollo docente en la EIP.
E16	Colaborativas^(15)^.	Aprendizaje experiencial.	Modelo de ejercicio de enseñanza estructurado por objetivos interprofesionales (iOSTE) y conferencias.	Instrumento cualitativo: evaluación por rúbricas y debriefing. Instrumento cuantitativo: cuestionarios. Aplicación: antes y después de la iniciativa para el desarrollo docente en la EIP. Instrumento cualitativo: evaluación por rúbricas y debriefing. Instrumento cuantitativo: cuestionarios. Aplicación: antes y después de la iniciativa para el desarrollo docente en la EIP.
E17	Mencionó pero no señaló un marco o estructura de competencias colaborativas.	No aparece.	Aprendizaje basado en proyectos.	Instrumento Cuantitativo cuestionario validado^(54)^. Aplicación: antes y después de la iniciativa para el desarrollo docente en la EIP.
E18	Colaborativas^(15)^.	Aprendizaje experiencial.	Seminarios, pósteres, mesa redonda temática con Aprendizaje activo, simulaciones basadas en casos y grupo de discusión colaborativo.	Instrumento cualitativo: cuestionarios. Aplicación: antes, el día de la actividad y al final de la iniciativa para el desarrollo docente en la EIP.
E19	Colaborativas^(14)^.	Aprendizaje experiencial.	Conferencias, talleres, seminarios, estudios de casos, juegos de roles, pacientes simulados, debates, pósteres, sesiones plenarias interactivas y sesiones de formación just-in-time.	Instrumentos cuantitativos: escalas validadas^(50,55)^. Aplicación: antes y después de la iniciativa para el desarrollo docente en la EIP.
E20	Colaborativas^(14,16)^.	Aprendizaje experiencial y significativa.	Actividades de aterrizaje check-in y check-out; grupos pequeños, círculo, cara a cara con interacción entre sí; escritura y lectura de investigación apreciativa, reflexión narrativa con mediación de un facilitador; reflexión basada en casos-ejercicios; Juego de roles enfoque interprofesional para practicar habilidades de comunicación de malas noticias y divulgación de errores y plan de acción para desarrollar competencias colaborativas^(47,48)^.	Instrumento cualitativo: cuestionario apreciativo con reflexión narrativa. Aplicación: tras la iniciativa para el desarrollo docente en la EIP.
E21	Colaborativas^(14,16)^.	Aprendizaje de adultos.	Diagnóstico situacional con el fin de explorar el potencial de éxito de la difusión de innovaciones en la institución de origen del docente, seminarios web, módulos interactivos en línea, capacitación para completar instrumentos de planificación, tutoría, retroalimentación, aprendizaje basado en problemas, informes de procesos y videos.	Instrumento cuantitativo: cuestionario validado^(56)^. Evaluación: antes y después de la iniciativa para el desarrollo docente en la EIP.
E22	Mencionó pero no señaló un marco o estructura de competencias colaborativas.	No aparece.	Workshop y actividades on-line y de simulación.	Instrumento cuantitativo: Escala validada^(50)^. Evaluación: antes y después de la iniciativa para el desarrollo docente en la EIP.
E23	Mencionó pero no señaló un marco o estructura de competencias colaborativas.	Citó sin dar detalles.	La escritura intencional/investigación como estrategia de aprendizaje para desarrollar nuevos facilitadores, currículos/actividades de EIP, aplicación de principios de aprendizaje activo, orientación y liderazgo.	Instrumentos cualitativos: cuestionarios para evaluar los cambios de comportamiento basados en la tipología de Kirkpatrick. Aplicación: los autores no lo han informado.
E24	Colaborativas^(14,16)^.	Aprendizaje de adultos.	Métodos interactivos (No se proporcionaron detalles).	No aparece.
E25	Colaborativas^(16)^.	Aprendizaje experiencial.	Sesiones en formato híbrido, actividades en grupos reducidos, estudio de casos y vídeos.	Instrumentos cualitativos: evaluación formativa, debriefing y grupos focales. Instrumentos cuantitativos: evaluación sumativa y cuestionarios basados en las competencias colaborativas interprofesionales^(16)^. Evaluación: antes y después de la iniciativa para el desarrollo docente en la EIP.
E26	Colaborativas^(16)^ y una competencia adicional: Humildad intercultural.	No aparece.	Actividades basadas en estudio de casos.	No aparece.

Legenda: *ID = Identificação do estudo.

Fonte: Elaborado pelas autoras.

**Figura 2:** Resumen de los resultados en las categorías de análisis - São Carlos, SP, Brasil, 2024.

**Fuente:** Figura elaborada por las autoras con la herramienta Canva.

En la primera categoría, las competencias identificadas incluyeron: competencias colaborativas interprofesionales y de facilitación para la EIP. Las competencias colaborativas interprofesionales para la EIP se abordaron de diversas maneras en las publicaciones basadas en referencias teóricas consolidadas^(14-16,47)^. El marco canadiense del CIHC^(14)^ se mencionó en siete estudios de esta revisión^(26,28,34,39-41,44)^. El marco estadounidense del IPEC^(15)^, publicado en 2011, se utilizó en seis estudios incluidos^(24,27,29,35,36,38)^, y su versión actualizada en 2016^(16)^ estaba presente en 10 estudios^(25,28,30,32,33,40,44,45,46)^. 

Solo un estudio^(23)^ utilizó el modelo de tres competencias para orientar la EIP: 1) Competencias comunes, conjunto de conocimientos generales para todas las profesiones; 2) Competencias complementarias o específicas, que diferencian una profesión de otra; y 3) Competencias colaborativas, entre profesionales de la misma profesión o de profesiones diferentes, con inclusión de usuarios, familias y comunidades^(47)^. Algunos estudios aplicaron ambos marcos^(14,16)^ para respaldar sus estudios^(28,40,41,44)^. Además, seis estudios informaron sobre la relevancia de las competencias colaborativas interprofesionales, pero no señalaron ningún marco o estructura para componer sus análisis^(13,22,31,37,42,43)^, y uno de ellos se centró en la comunicación^(22)^.

Entre las competencias colaborativas interprofesionales, 16 de los estudios incluidos destacaron los ámbitos del trabajo en equipo y la comunicación^(14, 15, 26, 28, 30, 33–36, 38, 41, 44–46)^ como parte relevante de las intervenciones realizadas, aunque no profundizaron en los detalles de los atributos y el rendimiento esperados.

Dos publicaciones^(23,35)^ incluyeron referencias teóricas sobre la facilitación en la EIP^(48)^ y las competencias de sensibilidad cultural y seguridad en la EIP^(49)^, aunque publicadas anteriormente a los cuadros/estructuras de competencias colaborativas, presentan características similares.

Las competencias para la facilitación de la EIP identificadas^(48)^ fueron: 1) Compromiso con la EIP y el PIC; 2) Credibilidad en relación con la EIP; 3) Modelado positivo de roles; 4) Comprensión profunda de los métodos de aprendizaje interactivo y confianza para su aplicación; 5) Conocimiento de las dinámicas de grupo; 6) Confianza y flexibilidad para utilizar las diferencias profesionales en grupo y 7) Equilibrio entre las necesidades individuales y las grupales, además de disposición ante las dificultades.

Las competencias de sensibilidad cultural y seguridad en la EIP^(49)^ se refieren a: 1) Realizar esfuerzos para modificar su perspectiva del mundo; 2) Comprender las cuestiones culturales fundamentales; 3) Comprender la cultura de los profesionales con los que trabaja; 4) Comprender las cuestiones culturales fundamentales que se refieren al proceso de salud y enfermedad; 5) Establecer una relación de confianza con los usuarios; 6) Proporcionar acogida en el contexto de la asistencia sanitaria; y 7) Negociar las intervenciones sanitarias, con el consentimiento de los usuarios.

La segunda categoría estaba compuesta por publicaciones que presentaban los fundamentos pedagógicos, teórico- metodológicos, teorías o enfoques de aprendizaje, estrategias de enseñanza-aprendizaje y evaluación adoptados por los docentes. De las 26 publicaciones incluidas, 15 abordaban teorías de aprendizaje para orientar las iniciativas de desarrollo docente en la EIP: aprendizaje experiencial^(22,23,30,34–36,38–40,45)^, aprendizaje de adultos^(35,41,44)^, aprendizaje significativo^(28,40)^, enfoque constructivista^(35)^, aprendizaje basado en competencias (enseñanza por competencias)^(13)^ y aprendizaje social^(32)^.

La mayoría de los estudios detallaron las estrategias de enseñanza y aprendizaje (Cuadro 3), aspecto ausente en una sola publicación, en la que tampoco se presentaron los instrumentos relativos al proceso evaluativo^(13)^. Además, 22 publicaciones describieron diferentes metodologías de enseñanza-aprendizaje, activas e interactivas, que favorecen el intercambio de experiencias entre docentes, supervisores, preceptores clínicos y profesionales de la salud^(22, 24–36, 38–45)^. Tres investigaciones mencionaron solo una estrategia de enseñanza-aprendizaje para la oferta pedagógica^(13,37,46)^.

Los estudios utilizaron una variedad de instrumentos cualitativos y cuantitativos para la evaluación, y diversificaron los períodos en que se aplicaron. Los instrumentos cuantitativos de evaluación se emplearon en ocho estudios, todos con dos momentos de aplicación, antes y después de las iniciativas para el desarrollo de la EIP^(24,26,27,30,37,39,41,42)^, entre los que se encuentran escalas tipo *Likert* y cuestionarios validados, como *The Interdisciplinary Education Perception Scale* ^(50)^, *The Interprofessional Education for Collaborative Patient-Centred Care Questionnaire* ^(51)^, *Interprofessional Collaborative Competencies Attainment Survey* ^(52)^, *Interprofessional Facilitation Scale* ^(53)^, *Perception of Interprofessional Collaboration Model Questionnaire* ^(54)^ *, Readiness for Interprofessional Learning Scale* ^(55)^ y *Core Competencies for Interprofessional Practice Individual Competency Assessment Tool* ^(56)^.

Los instrumentos cualitativos de evaluación fueron cuestionarios, entrevistas, ejercicios de reflexión, informes de grupo, evaluación por rúbricas, debates en pequeños grupos interprofesionales, *retroalimentación* presencial y autoevaluación. Seis estudios aplicaron instrumentos cualitativos para evaluar las iniciativas para el desarrollo docente en la EIP de forma aislada^(28,33,34,38,40,43)^. De los estudios analizados, tres no informaron sobre el momento de aplicación de los instrumentos de evaluación^(28,33,43)^ y dos los aplicaron solo después de la iniciativa^(34,40)^. Un estudio que aplicó el instrumento de evaluación solo al final no detalló los objetivos de aprendizaje, sin embargo, los participantes informaron reacciones positivas sobre la intervención, cambios de actitudes y percepciones, además de la adquisición de conocimientos y habilidades, de forma colaborativa^(34)^.

En total, siete estudios aplicaron instrumentos para evaluar los enfoques cualitativos y cuantitativos^(22,25,29,32,35,36,45)^. De estos, uno no describió el momento de su aplicación^(25)^, dos los aplicaron en cuatro momentos: antes, durante, al final y algún tiempo después de la intervención de EIP^(29,35)^, y uno de ellos aplicó la evaluación un año después de la intervención, aunque no se evaluaron sus beneficios^(29)^. Los demás estudios aplicaron instrumentos en dos momentos distintos, antes y después de la intervención^(22,32,36,45)^. 

## DISCUSIÓN

Esta revisión de alcance permitió mapear las competencias colaborativas interprofesionales que los docentes, supervisores, preceptores clínicos, profesionales de la salud y educadores consideran necesarias durante el proceso de formación interprofesional. También se identificaron los fundamentos pedagógicos y teórico-metodológicos abarcados por las teorías, los enfoques de aprendizaje, las estrategias de enseñanza-aprendizaje y de evaluación, que consisten en principios que facilitan el desarrollo de competencias para el desarrollo docente en la EIP. Se optó por sistematizar los fundamentos pedagógicos mencionados, considerando que el campo de la EIP tiene una especificidad que requiere la aplicación de estrategias activas, interactivas y compartidas de enseñanza-aprendizaje y evaluación. De este modo, los hallazgos pueden contribuir a dotar a los docentes de herramientas para el proceso de planificación, implementación y evaluación de propuestas curriculares e iniciativas de EIP.

Cabe destacar que la mayoría de las investigaciones identificadas utilizan los marcos CIHC e IPEC, lo que demuestra que sus iniciativas están alineadas con los marcos/estructuras de la PIC^(14–16)^. En la presente revisión se identificaron fuertemente los ámbitos de la comunicación y el trabajo en equipo, que expresan la intencionalidad de fortalecer la fuerza de trabajo en salud desde una perspectiva interprofesional. Sin embargo, es relevante destacar que el compromiso de la EIP también destaca la atención centrada en los usuarios, las familias y las comunidades, presente en estos marcos de referencia de análisis y aún incipiente en los estudios analizados.

En relación con la heterogeneidad de la formación en las iniciativas de desarrollo docente en EIP, deben tenerse en cuenta las particularidades y características de estos profesionales, como el régimen de trabajo a tiempo parcial o completo, las cualificaciones y responsabilidades docentes, condiciones que pueden influir en el enfoque y los métodos de facilitación en EIP^(35)^. Además, los docentes y otros facilitadores pueden tener dificultades para identificar oportunidades de aprendizaje en EIP en los escenarios de práctica, dados los diferentes repertorios y experiencias previas^(57)^. En este sentido, un estudio destacó que los supervisores y preceptores clínicos identificaron oportunidades para la PIC, sin embargo, los docentes no tuvieron la misma percepción y necesitaron trabajar conjuntamente con estos agentes en los escenarios prácticos para identificar y ampliar las oportunidades formativas^(34)^.

La literatura sugiere estrategias para hacer frente a estas dificultades de percepción de los docentes sobre las oportunidades reales de la EIP. La designación de agentes con experiencia en la facilitación de la EIP puede proporcionar oportunidades para experimentar diferentes métodos de enseñanza-aprendizaje y dinámicas de trabajo en equipo^(35)^. La participación de agentes con experiencia en EIP puede contribuir a la difusión de sus fundamentos pedagógicos y supuestos, a la valorización de los diferentes conocimientos interprofesionales y al fortalecimiento de las asociaciones en la integración enseñanza-servicio-comunidad, orientadas a la construcción de experiencias y reflexiones con énfasis en la EIP, desde el inicio del recorrido formativo.

Se han podido identificar y sintetizar teorías, enfoques de aprendizaje, estrategias de enseñanza-aprendizaje y de evaluación acordes con la EIP. Como también se destaca en los resultados de esta revisión, una investigación reveló que muchas intervenciones pedagógicas en EIP no presentaban detalles precisos sobre la teoría de aprendizaje elegida para guiar las intervenciones^(58)^. Esto puede deberse a la falta de comprensión de las teorías de aprendizaje empleadas en las iniciativas de EIP, pero también puede estar asociado a la falta de directrices pedagógicas, objetivos, resultados de aprendizaje y análisis del impacto de las iniciativas de EIP en el profesorado^(59,60)^. La ausencia de estas teorías o enfoques pedagógicos puede dificultar la construcción y la implementación de iniciativas exitosas de desarrollo docente en EIP.

Las teorías del aprendizaje seleccionadas en los estudios se aplicaron de forma independiente o combinada. Tres estudios utilizaron más de una teoría del aprendizaje para fundamentar las prácticas pedagógicas, pero no describieron su articulación con los demás componentes pedagógicos ni sus impactos^(32,35,40)^. Las teorías del aprendizaje son aproximaciones parciales y restringidas de aspectos y áreas específicas del aprendizaje, por lo que difícilmente constituyen un conjunto completo de conocimientos que aclaren el significado de los fenómenos complejos que se producen durante el aprendizaje^(61)^. Si una teoría o enfoque no es suficiente para comprender los complejos fenómenos del aprendizaje, se puede inferir que la planificación pedagógica debe basarse en más de una teoría o enfoque, de modo que pueda satisfacer las diversas necesidades de los estudiantes durante las actividades de EIP.

La teoría del aprendizaje de adultos destaca la responsabilidad y el protagonismo del sujeto en su propio aprendizaje, que en el caso de la EIP también se comparte entre personas y grupos. Los principios de la educación de adultos enfatizan el aprendizaje cooperativo, colaborativo, reflexivo y socialmente construido entre personas^(62)^, en consonancia con los principios y valores de la EIP, que incluyen la colaboración, la interacción, el respeto mutuo, las asociaciones y la responsabilidad^(63)^.

Las experiencias para el desarrollo docente en la EIP también revelaron el enfoque constructivista, en el que el ser humano es visto como alguien que se transforma a través de las relaciones e interacciones con el entorno social y cultural en el que se encuentra. Es decir, el aprendizaje y la transformación a través de las interacciones sociales^(64)^. El enfoque constructivista puede ser la base para la elaboración, la oferta y el proceso de evaluación de las iniciativas de desarrollo docente en la EIP, ya que los estudiantes y los profesionales de la salud necesitan relacionarse constantemente. Por lo tanto, es necesario priorizar e incentivar las experiencias de aprendizaje que propicien las interacciones interprofesionales, entre profesionales, estudiantes^(35,44)^ y estos con los usuarios, las familias y las comunidades.

Otra perspectiva presentada en los resultados fue la del aprendizaje experiencial, en el que el conocimiento se construye y modifica a través de las experiencias. De este modo, el conocimiento no se considera un elemento inmutable, como en el enfoque tradicional. La experiencia es el resultado de la interacción entre el mundo exterior y el interior del ser, a partir de situaciones que permiten la creación y la recreación del conocimiento^(65)^. Durante las experiencias, en la construcción y reconstrucción de nuevos conocimientos, pueden surgir conflictos que dan lugar a una mejora de las percepciones, la comprensión y la adquisición de nuevas competencias. En este proceso, los participantes de la comunidad de aprendizaje trabajan juntos en la comprensión de los fenómenos, la toma de decisiones y la resolución de problemas^(66)^, aspectos que refuerzan la necesidad de experiencias de EIP en escenarios reales de prácticas de salud.

Las publicaciones analizadas también trataron sobre enfoques activos e interactivos, dada la importancia del intercambio intencional entre estudiantes, educadores, supervisores, preceptores y docentes^(44,59)^, que se alinean con los principios y valores de la EIP. La metodología activa e interactiva amplía las posibilidades de enseñanza y aprendizaje crítico-reflexivo, el compromiso y el protagonismo del estudiante, las oportunidades para mejorar las competencias colaborativas^(63)^ y hacer que el aprendizaje sea más atractivo y eficaz^(58)^.

En resumen, la mayoría de las investigaciones mencionaron las competencias colaborativas interprofesionales que ofrece el enfoque experiencial, entre otras teorías y fundamentos pedagógicos, de forma aislada o complementaria, como el aprendizaje significativo y socio-constructivista de adultos. El nexo común entre estos enfoques es el desplazamiento del protagonismo en el aprendizaje, del docente al estudiante.

La literatura recomienda que las experiencias de formación profesional en salud presenten las teorías que las fundamentan de forma más explícita y sólida, el objetivo de la educación, la naturaleza del conocimiento, lo que se valora e incluye en el currículo, el significado de aprender y cómo se evalúa, los roles de profesores y estudiantes en el proceso, es decir, que haya alineación entre las prácticas de enseñanza, aprendizaje y evaluación con los valores y supuestos paradigmáticos^(67)^. Hay incluso investigaciones que indican el avance de la teorización de la práctica de la enseñanza-aprendizaje imbuida de emociones, con el reconocimiento de que la educación va más allá del cognitivismo, es un proceso afectivo, social y político^(68)^. Otro estudio destacó la dificultad de los docentes para presentar previamente la teoría que sustenta las experiencias, indicada solo a posteriori^(69)^. En una revisión sobre las intervenciones de EIP, se indicó un conjunto de recomendaciones para que los educadores diseñen, implementen y evalúen la EIP, con el fin de alcanzar las competencias interprofesionales. Específicamente en relación con el desarrollo docente, objeto de interés de esta revisión, se identificó que estos reciben formación antes de participar en la intervención, pero no hay información sobre si es específica para la facilitación de la EIP o de naturaleza pedagógica general^(70)^.

Los estudios anteriores^(67-70)^ refuerzan los hallazgos de esta revisión: fragilidades entre los principios teóricos y la práctica docente, experiencias empíricas en detrimento de una reflexión teórica más profunda, falta de consenso sobre qué teorías están en el origen de la EIP y la PIC y cómo aplicarlas. Las dificultades enumeradas expresan la complejidad en la construcción de experiencias formativas de EIP, razones que también pueden estar relacionadas con la escasa presencia de teorías y fundamentos teóricos en las iniciativas de desarrollo docente en EIP.

En relación con el proceso evaluativo, el Centro para el Avance de la Educación Interprofesional (CAIPE, por sus siglas en inglés) propone la implementación de estrategias dinámicas, diversificadas y continuas a lo largo de la acción en EIP^(63)^. El proceso de evaluación debe pensarse en la elaboración del proyecto, con la definición de los instrumentos utilizados, alineados con los objetivos, los resultados del aprendizaje y los momentos de su aplicación^(71)^.

En lo que respecta a la articulación de la EIP para el fortalecimiento de las prácticas de los sistemas de salud, que en Brasil se refiere al SUS, aunque ninguno de los estudios se ha realizado en el sistema de salud brasileño, las evidencias infieren la responsabilidad de las Instituciones de Educación Superior (IES), a través de políticas y programas de desarrollo docente, cuyo horizonte es la mejora de los resultados en salud, desde la perspectiva de la interdependencia entre el sistema de salud y el de educación. El compromiso social de las IES reside en la formación de una fuerza de trabajo preparada para atender las necesidades de los usuarios y de los sistemas de salud, con énfasis en la calidad. La priorización de los docentes y facilitadores en el desarrollo de competencias colaborativas interprofesionales permite fortalecer el trabajo en equipo, la colaboración interprofesional y la atención centrada en los usuarios.

A pesar del reto que supone la formación en competencias colaborativas interprofesionales, integradas a las necesidades de las personas, las familias y las comunidades, y del reconocimiento de la importancia de avanzar en esta dirección, en el panorama brasileño sigue predominando la oferta de cursos que no incluyen períodos en el plan de estudios para la EIP y, cuando lo hacen, se trata de actividades esporádicas y desde una perspectiva multiprofesional^(72)^.

La formación docente para la EIP tiene el potencial de estimular el reconocimiento de las oportunidades de formación interprofesional en contextos de práctica, de oportunidades relevantes, que impacten en el conocimiento, las habilidades y la confianza en el proceso de enseñanza-aprendizaje^(73)^, cuyo punto de partida es la existencia de planes de estudio más flexibles y articulados.

Aunque el estudio ha cumplido con los procedimientos metodológicos recomendados para una revisión de alcance, con un amplio mapeo de la literatura y sin restricciones temporales, es importante considerar sus limitaciones. La restricción de la búsqueda a tres idiomas, portugués, inglés y español, puede haber provocado pérdidas, especialmente de publicaciones en otros idiomas. Solo se incluyó en los resultados una publicación identificada como literatura gris, lo que puede ser consecuencia de imprecisiones en el mapeo de este tipo de publicaciones. La heterogeneidad de los diseños de las publicaciones incluidas para la elaboración de síntesis y la falta de evaluación de la calidad metodológica de los estudios incluidos, etapa poco presente en las revisiones de alcance.

## CONCLUSIÓN

Esta revisión de alcance tuvo como objetivo mapear las competencias de desarrollo docente para la EIP, lo que requirió el análisis de las competencias colaborativas interprofesionales y de facilitación presentes en las intervenciones educativas, con énfasis en los ámbitos de la comunicación y el trabajo en equipo.

Las evidencias producidas destacan la necesidad de futuros estudios sobre el desarrollo de docentes y facilitadores de la EIP con énfasis en la integración enseñanza-servicio-comunidad, así como en la construcción, difusión y aplicación de un marco de competencias desde la perspectiva pedagógica en la EIP. También se necesitan avances en investigaciones que demuestren las articulaciones entre las teorías/enfoques pedagógicos y las competencias para el desarrollo docente en la EIP, así como los resultados de las evaluaciones posteriores a las iniciativas, sobre todo a largo plazo.

En la presente revisión del alcance se concluye que los ámbitos de competencias para el desarrollo docente en la EIP pueden dividirse en dos grupos: 1) Competencias colaborativas interprofesionales y de facilitación para la EIP y 2) Fundamentos pedagógicos, teórico-metodológicos para el desarrollo docente en la EIP: teorías, enfoques de aprendizaje, estrategias de enseñanza-aprendizaje y de evaluación. El presente mapeo contribuye a la fundamentación teórica en la planificación, implementación y evaluación de iniciativas para el desarrollo de la EIP y de sus agentes, docentes, supervisores clínicos, preceptores de los escenarios de práctica, profesionales de la salud y los propios estudiantes, con el fin de fortalecer la interprofesionalidad.

A partir de los resultados, se recomienda que los gestores de cursos de salud, los coordinadores de programas de desarrollo docente y los formuladores de políticas educativas reconozcan la importancia de las teorías del aprendizaje en el fortalecimiento de la EIP y el desarrollo docente, con la recomendación de que estos fundamentos/teorías pedagógicas se expliquen y estén alineados y sean coherentes con las características de la EIP. Además, se recomienda invertir en espacios formativos, con amplia participación de los diferentes agentes corresponsables de la formación en salud, además del cuerpo docente.

## DISPONIBILIDAD DE DATOS

Todo el conjunto de datos que respalda los resultados de este estudio está disponible previa solicitud al autor correspondiente [Jaqueline Alcântara Marcelino da Silva].

## Footnotes

1 En esta publicación se consideran docentes aquellos que planifican, implementan y evalúan las acciones curriculares de la formación en salud y facilitadores, incluyendo los demás agentes involucrados en la formación en salud, tales como preceptores, supervisores, tutores, mentores que no siempre participan en todas las etapas de los procesos formativos.
